# Dimethyl itaconate ameliorates the deficits of goal-directed behavior in *Toxoplasma gondii* infected mice

**DOI:** 10.1371/journal.pntd.0011350

**Published:** 2023-05-31

**Authors:** Yongshuai Wu, Daxiang Xu, Yan He, Ziyi Yan, Rundong Liu, Zhuanzhuan Liu, Cheng He, Xiaomei Liu, Yinghua Yu, Xiaoying Yang, Wei Pan

**Affiliations:** 1 Jiangsu Key Laboratory of Immunity and Metabolism, Jiangsu International Key Laboratory of Immunity and Metabolism, Department of Pathogen Biology and Immunology, Xuzhou Medical University, Xuzhou, China; 2 The First Clinical Medical College, Xuzhou Medical University, Xuzhou, China; 3 National Experimental Teaching Demonstration Center of Basic Medicine (Xuzhou Medical University), Xuzhou, China; Indian Institute of Science Education and Research Bhopal, INDIA

## Abstract

**Background:**

The neurotrophic parasite *Toxoplasma gondii* (*T*. *gondii*) has been implicated as a risk factor for neurodegenerative diseases. However, there is only limited information concerning its underlying mechanism and therapeutic strategy. Here, we investigated the effects of *T*. *gondii* chronic infection on the goal-directed cognitive behavior in mice. Moreover, we evaluated the preventive and therapeutic effect of dimethyl itaconate on the behavior deficits induced by the parasite.

**Methods:**

The infection model was established by orally infecting the cysts of *T*. *gondii*. Dimethyl itaconate was intraperitoneally administered before or after the infection. Y-maze and temporal order memory (TOM) tests were used to evaluate the prefrontal cortex-dependent behavior performance. Golgi staining, transmission electron microscopy, indirect immunofluorescence, western blot, and RNA sequencing were utilized to determine the pathological changes in the prefrontal cortex of mice.

**Results:**

We showed that *T*. *gondii* infection impaired the prefrontal cortex-dependent goal-directed behavior. The infection significantly downregulated the expression of the genes associated with synaptic transmission, plasticity, and cognitive behavior in the prefrontal cortex of mice. On the contrary, the infection robustly upregulated the expression of activation makers of microglia and astrocytes. In addition, the metabolic phenotype of the prefrontal cortex post infection was characterized by the enhancement of glycolysis and fatty acid oxidation, the blockage of the Krebs cycle, and the disorder of aconitate decarboxylase 1 (ACOD1)-itaconate axis. Notably, the administration of dimethyl itaconate significantly prevented and treated the cognitive impairment induced by *T*. *gondii*, which was evidenced by the improvement of behavioral deficits, synaptic ultrastructure lesion and neuroinflammation.

**Conclusion:**

The present study demonstrates that *T*. *gondii* infection induces the deficits of the goal-directed behavior, which is associated with neuroinflammation, the impairment of synaptic ultrastructure, and the metabolic shifts in the prefrontal cortex of mice. Moreover, we report that dimethyl itaconate has the potential to prevent and treat the behavior deficits.

## Author summary

*Toxoplasma gondii* (*T*. *gondii*) is a neglected but important risk factor for neurodegenerative diseases. It is necessary to decipher the mechanism of cognitive deficits induced by the parasite and develop intervention therapy. Here, we showed that chronic *T*. *gondii* infection induces the deficits of the goal-directed behavior, which is associated with the prefrontal cortex. Moreover, the global expression pattern in the brain area of the infected mice is characterized by the downregulation of the genes associated with synaptic transmission and plasticity, and the upregulation of the genes related to neuroinflammation including the activation makers of microglia and astrocytes. Interestingly, the ACOD1-itaconate axis, a key node modulating immunity and metabolism, is activated post infection. We reported that dimethyl itaconate (the derivative of itaconate) effectively prevents and treats the goal-directed cognitive deficits induced by *T*. *gondii*, which was proved by the improvement of the synaptic ultrastructure impairment and neuroinflammation. Overall, these findings provide a novel insight for understanding the mechanism of cognitive deficits induced by *T*. *gondii* and developing a therapeutic strategy against the parasite-related neurodegenerative diseases.

## Introduction

Neurodegenerative diseases, such as Alzheimer’s disease (AD), characterized by cognitive decline and neuroinflammation, cause a heavy economic burden worldwide [1, 2]. However, few effective drugs are currently available for treatment [3]. *Toxoplasma gondii* (*T*. *gondii*) is a neurotropic parasite with an exceptionally broad host range affecting approximately 30% of the global population [4]. *T*. *gondii* infection can be categorized into acute infection and chronic infection. It is well-established that acute infection can be lethal and malformed to the fetus during pregnancy [5]. However, the consequence of chronic infection (a stage when the parasite forms cysts, mainly located in the brain of immune-competent individuals [6]), has been largely neglected due to slight clinical performance. Interestingly, several studies have provided evidence for the cognitive and behavioral changes induced by the chronic infection of the parasite. In humans, chronic *T*. *gondii* infection is reported to have a positive correlation with several mental illnesses, including epilepsy, AD, schizophrenia, and bipolar disorder [7–9]. In line with this, *T*. *gondii*-infected mice exhibit poor learning and memory ability compared with control mice [10, 11]. Moreover, two recent studies have shown that the infection can downregulate the expression of synapse-associated proteins and spur the signs of AD in mice [12, 13]. Overall, these findings indicate that *T*. *gondii* infection can cause cognitive deficits and act as one of the risk factors for neurodegenerative diseases.

It is well known that immune modulation plays a pivotal role in the homeostasis of the central nervous system under physiological conditions [14]. However, chronic neuroinflammation can lead to neurodegenerative diseases [14, 15]. In the animal models of AD, activated microglia and astrocytes not only lead to the loss of synaptic components and decreased synaptic plasticity [16] but also release pro-inflammatory cytokines including interleukin-1α (IL-1α), interleukin-1β (IL-1β) and interleukin-6 (IL-6), thereby accelerating the amyloid β (Aβ) deposition and cognitive decline [17]. Therefore, modifying neuroinflammation is considered to be a promising treatment strategy against cognitive deficits associated diseases [18]. In recent years, a battery of studies has shown that neuroinflammation is a crucial mechanism of *T*. *gondii* infection-induced odd behaviors [19, 20]. However, it is still unknown if remolding neuroinflammation could improve *T*. *gondii*-induced cognitive impairment.

Immunometabolism, an emerging field in recent years, highlights the profound impact of metabolic reprogramming on immune function [21]. In the resting state, microglia featured with neuroprotective role conduct oxidative phosphorylation (OXPHOS) [22]. However, in the pathological state, microglia polarize towards the M1 phenotype [23]. Such change can prompt the transformation of resting astrocytes into the A1 phenotype, thus promoting the progression of neurodegenerative diseases [24, 25]. To initiate and maintain intact function, the inflammatory microglia undergo metabolic reprogramming from OXPHOS to anaerobic glycolysis [26]. Interestingly, interferon-gamma (IFN-γ) supplementation can restore the function of microglia via reprogramming metabolic profile, thereby alleviating the symptoms of AD [27]. Thus, reprogramming microglia metabolism represents a novel direction for designing novel therapies against neuroinflammation and neurodegenerative diseases.

Aconitate decarboxylase 1 (ACOD1), also known as immune responsive gene 1 (IRG1), is an enzyme in the Krebs cycle (tricarboxylic acid cycle, TCA cycle), which catalyzes the conversion of cis-aconitate to itaconate. It is well established that the ACOD1-itaconate axis is a key node modulating immunity and metabolism in macrophage [28]. Itaconate is shown to activate anti-inflammatory nuclear factor erythroid 2-related factor 2 (Nrf2) in lipopolysaccharide (LPS)-induced macrophage inflammation [29]. In addition, itaconate is reported to inhibit succinate dehydrogenase (SDH) activity, thereby suppressing the inflammation mediated by succinate oxidation [30]. Notably, it has been reported that glycolysis, a primary pro-inflammatory metabolic phenotype in activated macrophage, can be downregulated by itaconate and its derivatives [31, 32]. Recently, dimethyl itaconate (DI), a cell-permeable itaconate derivative, exhibits immunomodulatory effects on neuroinflammation in the mouse model of experimental autoimmune encephalomyelitis (EAE) [33]. Intriguingly, mice primed and reinfected with *T*. *gondii* have high gene expression of ACOD1 in microglia [34]. These findings prompt us to investigate whether itaconate delivery could ameliorate the cognitive impairment induced by *T*. *gondii* infection by modulating neuroinflammation.

Different areas of the brain manipulate differential behaviors. The prefrontal cortex is a region at the anterior end of the brain. This area contributes to cognition controlling abilities by providing a structural basis for complex goal-directed behavior [35, 36] and can be assessed by behavioral tests such as Y-maze alternation [37] and temporal order memory [38]. Normal goal-directed behavior requires appropriate attentional, decision-making, and coordinative functions [35]. Notably, in a neuropsychological test, elderly individuals with *Toxoplasma* seropositivity exhibit the delaying processes of attention and disengagement [39]. Interestingly, Boillat *et al*. reported that the highest cyst density occurs in the cortex of *T*. *gondii*-infected mice [19], suggesting that the parasite may have a tropism for the cortex. Overall, these results indicate *T*. *gondii* chronic infection can compromise prefrontal cortex-associated cognition and behavior.

In the present study, we assessed the effect of *T*. *gondii* chronic infection on the goal-directed cognitive behavior and the neuropathological changes in the prefrontal cortex (PFC) of mice. Moreover, we evaluated the preventive and therapeutic role of dimethyl itaconate, a derivative of itaconate, in the deficits of the goal-directed behavior induced by the parasitic infection. Overall, this study provides a novel insight for clarifying the underlying mechanisms of how *T*. *gondii* induces the cognitive impairment and proposes that DI is a promising drug candidate against *T*. *gondii-*related neurodegenerative diseases.

## Methods and materials

### Ethics statement

The animal study was reviewed and approved by the Ethics Committee of Xuzhou Medical University (Xuzhou, China, SCXK (Su) 2020–0048).

### Animals and parasite

C57BL/6J mice (7 weeks old) were purchased from Jiangsu Jicui Pharmaceutical Technology Corporation (Jiangsu Province, China), and bred in a pathogen-free environment in the university institute. All mice were housed in an air-conditioned room at 24°C with a 12 h dark /light cycle and permitted free access to food and water. *T*. *gondii* cysts (wh6 isolate, a low virulent strain and usually causes chronic infection [40]), isolated from the brains of mice with *T*. *gondii* infection, were used to establish the infection model.

### Establishment of chronic infection and Dimethyl itaconate administration

There are 3 experiments in the present study. Experiment 1 aimed to evaluate the effect of *T*. *gondii* chronic infection on cognitive function. Mice were randomly separated into 2 groups: (a) Mice received phosphate buffer saline (PBS) by gavage as control (Con) group; (b) Mice received *T*. *gondii* cysts by the gavage (10 cysts for each mouse) as Tg group. The detailed process of infection was carried out as previously reported [41].

Experiment 2 aimed to determine the preventive effect of Dimethyl itaconate (DI) on *T*. *gondii*-induced abnormal behavior and neuropathologic lesion in the prefrontal cortex. Mice were randomly separated into 4 groups: Con and Tg groups were treated as mentioned in Experiment 1. In the Con+Veh group, mice received PBS as vehicle control. In the Con+ DI group, mice received 40 mg DI (No. 617527, Sigma-Aldrich, St. Louis, MO, USA) per kilogram body weight. In the Tg + Veh group, the *T*. *gondii-*infected mice were received with PBS. In the Tg + DI group, each *T*. *gondii-*infected mouse received 40 mg DI per kilogram body weight. DI administration (intraperitoneal injection, twice per week) started one week before *T*. *gondii* infection and lasted until the end of the experiment.

Experiment 3 aimed to evaluate the therapeutic effect of DI on *T*. *gondii-*induced behavior deficits. Mice were randomly separated into 3 groups: Con+Veh, Tg+Veh, and Tg + DI groups. Con and Tg groups were treated as mentioned in Experiment 1. Four weeks after infection, the infected mice were intraperitoneally injected with DI (40 mg/kg) as the Tg+DI group, while the infected mice received PBS as the Tg+Veh group. DI administration (twice per week) lasted until the end of the experiment.

All mice were sacrificed 4 days after behavioral tests with CO_2_. The prefrontal cortex tissues were collected for further analyses.

### Y-Maze test

The Y-maze test was performed to assess spatial working memory in mice [42]. In the test, a three-arm Y-maze with equal angles between all arms was used. Each mouse was placed in the center of the maze and allowed to explore freely during an 8-min session. The sequence and the total number of arms entered were recorded manually. A spontaneous alternation was defined as entries into three different arms consecutively (i.e., 123, 231, or 321, but not 212 and 323). An arm entry was considered to be complete when the hind paws of the mouse were completely within the arm. A mouse with intact working memory, and hence intact prefrontal cortical functions, will remember the arms previously visited and show a tendency to enter a less recently visited arm. The sessions were filmed by a video camera and the Y-maze arms were thoroughly cleaned with 70% ethanol to remove residual odors after each test. The calculation was defined as follows: percentage alternation = (number of alterations) / (total number of entries—2) × 100% [43]. A greater percentage of spontaneous alternation behavior reflected improved cognitive function [44]. The number of total arm entries served as an indicator of locomotor activity.

### Temporal order memory (TOM) test

The temporal order memory test assesses recognition memory processes based on relative recency information [45]. The test, which was divided into three secessions including habituation, sample secession, and test secession, was conducted for 2 days according to previously described methods [46]. On the first day, mice were acclimated to the room containing the behavioral facilities for 60 min. On the second day, each mouse underwent two sessions exploring two unique sets of objects (Objects A, B). During the test session, the mouse was allowed to explore an unused copy of Object A and an unused copy of Object B for 3 min. Mouse sniffing or touching the object with its nose, vibrissa, mouth, or forepaws was considered the time spent exploring the object. Intact temporal order memory is evident when mice spend more time exploring the old object (Object A) than the relative novel object presented (Object B). The discrimination index was determined according to the following equation: (T_old_—T_novel_) /(total exploration time) × 100% [47].

### Transmission electron microscope

Mice were sacrificed 4 days after behavioral tests with CO_2_. The prefrontal cortex was taken and rapidly fixed in glutaraldehyde. After fixation for 24 h, the cortical tissues were quickly dissected and separated into thin sections. They were fixed immediately with 2.5% glutaraldehyde at 4°C overnight. Washed 3 times in PBS, these sections were fixed in 1% osmium tetroxide, stained with 2% aqueous solution of uranyl acetate, and then dehydrated with different concentrations of ethanol and acetone gradient. Finally, they were embedded in epoxy resin. Ultra-thin sections (70 nm) were cut with ultramicrotome, collected on copper grids, and then stained with 4% uranyl acetate and 0.5% lead citrate. The ultrastructure of synapses in the PFC was photographed under a transmission electron microscope (HT7800, Hitachi, Japan) Synapses are classified into asymmetric and symmetric synapses, or Gray I type and Gray II type synapses, which are considered to mediate excitatory and inhibitory transmission, respectively. Asymmetric synapses have prominent postsynaptic densities and relatively wide synaptic clefts while symmetric synapses are with pre- and postsynaptic densities of equal thickness and narrower synaptic clefts. In this study, asymmetric synapses were examined for excitatory synaptic measurement. The software ImageJ (Version 1.53n, https://imagej.nih.gov/ij/) was used to assess the microstructural parameters including the postsynaptic density (PSD) thickness (measured the length of a perpendicular line traced from the postsynaptic membrane to the most convex part of the synaptic complex), the widths of the synaptic clefts (SC) (evaluated the widest and narrowest portions of the synapse and then averaging these values), the length of the active zone (AC), the synaptic curvature (estimated the ration of synaptic post interface arch length and chord length).

### Western blotting

Tissues of the prefrontal cortex were homogenized in ice-cold RIPA lysis buffer, supplemented with EDTA, protease inhibitor cocktail, and phosphatase inhibitor. The homogenate was sonicated six times for 4 seconds, at 6 seconds intervals on ice, and then centrifuged at 3864 *g* for 20 min at 4°C. The supernatant was collected, and the protein concentration was quantitated by BCA assay (Beyotime, P0010). Equal amounts of protein were separated by sodium dodecyl sulfate-polyacrylamide gel electrophoresis (SDS-PAGE) and transferred onto polyvinylidene difluoride (PVDF) membranes. The membrane was blocked with 5% non-fat milk (2.5 g skim milk powder + 50 ml washing buffer) at room temperature for 1 h and then incubated with the primary antibody at 4°C overnight. These primary antibodies included: rabbit anti-synaptophysin (Abcam, ab32127), rabbit anti-PSD95 (Invitrogen, 51–6900), and β-actin (ABclonal, AC026). Following 3 washes in TBST, the membrane was incubated with HRP-inked anti-rabbit IgG secondary antibody (CST, 7074) at room temperature for 1 h. After washing 3 times with TBST, the protein bands were detected with ECL western blot substrate (Bio-Rad, 1, 705,060) and visualized using the ChemiDoc Touch imaging system (Bio-Rad).

### Golgi staining

For detailed characterization of the neuronal processes and spines, we performed Golgi staining using the FD Rapid Golgi Stain Kit (PK401, FD NeuroTechnologies, Ellicott City, MD) as described in detail previously [48]. Briefly, the separated brains were soaked in a mixture of solution A and solution B prepared earlier in the dark for two weeks. Changed the mixture the next day. The tissue was then transferred to solution C, soaked for 72 hours, and the solution was replaced 24 hours later. The sections were obtained by a vibratome (100 μm) and placed in the mixture of solution D, solution E, and double distilled water for 10 minutes. Rinse sections with distilled water twice for 4 minutes each time. Sections were dehydrated in 50%, 75%, and 95% ethanol for 4 minutes each time and then in anhydrous ethanol 4 times for 4 minutes each time. Transparent in xylene for 4 minutes each time. The tablets were sealed with resin. Dry in the dark and take pictures using an Olympus microscope. Pyramidal neurons in layers II/III of the prefrontal cortex were analyzed. The Neuron J plugin of ImageJ software was used to track the neurite of a neuron and calculate the neuron’s total neurite length, length per neurite, and number of neurites per neuron. For the quantification of dendritic spines, we estimated the spine density by counting the number of spines along a section of the shaft. The spines were counted in a blind manner using the Cell Counter plugin of ImageJ software.

### Sholl analysis

Sholl analysis was performed using the Sholl plugin of ImageJ software to describe the morphology of neurons as previously reported [49]. The images of Golgi-stained pyramidal neurons were overlaid with concentric circles with a radial interval of 10 μm between each circle. The minimum radius is 10 μm and the maximum radius is 300 μm. The center point of the circles was superimposed over the cell body of previously acquired Golgi-stained neurons, and the number of neurites crossing each circle was manually counted. The morphology of the neuron is described by the number of intersecting points between the neurite and each circle, and the following indicators were calculated: sum intersections, and max intersection distance.

### Immunofluorescence

Mice in each group were transcardially perfused first with PBS and then fixed in a 4% paraformaldehyde (G1101, Servicebio, China) solution at room temperature before organ collection. The prefrontal cortex was removed from the brain, post-fixed overnight in 4% PFA, and then dehydrated in 30% sucrose solution for 2 days at 4°C. The harvested PFC were sectioned to 3 μm thickness in a rotary microtome (RM2016, Leica, German). The sections were blocked with BSA (G5001, Servicebio, China) for 30 min at room temperature and incubated with primary antibody rabbit anti-interleukin-6 (IL-6, 1:200, Servicebio) overnight at 4°C. Then the sections were incubated with HRP-inked goat anti-rabbit IgG secondary antibody (gb21303, 1:300, Servicebio) or Cy3 conjugated goat anti-mouse IgG secondary antibody (gb25303, 1:300, Servicebio) for 50 min at room temperature. Then the sections were incubated with FITC-TSA (G1222, 1:1000, Servicebio) at room temperature for 10 min. After incubation, the sections were washed with TBST 3 times, 5 min each. To remove the bound primary and secondary antibodies, the tissue sections were placed in the repair box filled with EDTA antigen repair buffer (G1206, Servicebio) and heated in the microwave oven. The second primary antibodies anti-calcium-binding adapter molecule 1 (Iba1, Ab178847, 1:100, Abcam) and mouse anti-glial fibrillary acidic protein (GFAP, #3670, 1:300, CST) were dropped onto the sections and incubated at 4°C overnight. The sections were washed with PBS 3 times, 5 min each time. Then, the sections were incubated with Cy3 conjugated goat anti-rabbit IgG secondary antibody (gb21303, 1:300, Servicebio) at room temperature for 50 min. The images were captured using a fluorescence microscope (Eclipse C1, Nikon, Japan). As described previously [50–52], the number of Iba1^+^ cells of microglia, GFAP^+^ cells of astrocyte, percentage of Iba1^+^IL-6^+^ cells were calculated using ImageJ. For cell morphology analysis, the circularity and solidity of Iba1^+^ cells were quantified by ImageJ. The circularity and solidity were defined as 4π × ([Aera]/[Perimeter]^2^) and [Aera]/[Convex area], respectively.

### Real-time PCR

Total RNA was extracted from tissues homogenized in Trizol (Thermo Fisher Scientific, Waltham, MA, USA). One microgram of purified RNA was used for RT-PCR to generate cDNA with a High-Capacity cDNA Reverse Transcription Kit (Takara, Dalian, China), and the resulting cDNA was used for quantitative PCR on a real-time PCR detection system (Bio-Rad, Hercules, CA, USA). The relative mRNA expression level was determined with the 2^-ΔΔCt^ method with β-actin as the internal reference control. Primer sequences were listed in S1 Table.

### Cyst burden counting

The brain tissues from Tg+Veh and Tg+DI group mice were homogenized in 1 ml PBS. The cyst burden was evaluated based on a previous protocol [12]. Briefly, 10 μl brain suspension was screened via light microscopy (×20). The cyst number was counted blindly to estimate the total cyst burden in the brain tissue. The counting process for each mouse was repeated 4 times.

### Genome-wide RNA Sequencing (RNA-seq)

Six weeks after the *T*. *gondii* infection, the prefrontal cortex was dissected under RNase-free conditions. Total RNA was extracted from the tissue using a Trizol reagent kit (Invitrogen, Carlsbad, CA, USA) according to the manufacturer’s protocol. RNA quality was assessed on an Agilent 2100 Bioanalyzer (Agilent Technologies, Palo Alto, CA, USA) and checked using RNase-free agarose gel electrophoresis. After total RNA was extracted, eukaryotic mRNA was enriched by Oligo(dT) beads, while prokaryotic mRNA was enriched by removing rRNA by Magnetic Kit (Epicentre, Madison, WI, USA). Then the enriched mRNA was fragmented into short fragments using a fragmentation buffer and reverse transcripted into cDNA with random primers. Second-strand cDNA was synthesized by DNA polymerase I, RNase H, dNTP, and buffer. Then the cDNA fragments were purified with a QiaQuick PCR extraction kit(Qiagen, Venlo, The Netherlands), end-repaired, poly(A) added, and ligated to Illumina sequencing adapters. The ligation products were size selected by agarose gel electrophoresis, PCR amplified and sequenced using Illumina HiSeq2500 by Gene Denovo Biotechnology Corporation (Guangzhou, China). DEGs were assessed by analysis of differential RNA expression between 2 groups. Transcripts with the parameter of a *P* value below 0.05 and an absolute fold change of 2 or greater were considered differentially expressed. Gene ontology (GO) and Kyoto Encyclopedia of Genes and Genomes (KEGG) Pathway enrichment analysis was performed using the DAVID Bioinformatics Resources 6.8 (https://david.ncifcrf.gov/) [53]. *P* value of 0.05 or less was considered as a threshold. Pathways meeting this condition were defined as significantly enriched pathways in DEGs. For quantification of gene abundance, an FPKM (fragment per kilobase of transcript per million mapped reads) value was calculated to quantify its expression abundance and variations, using StringTie software v1.3.1 [54, 55].

### Gene Set Enrichment Analysis (GSEA)

Traditional strategies for gene expression analysis have focused on identifying individual genes that exhibit differences between two states of interest. Although useful, they tend to ignore certain significant biological processes that are distributed across an entire network of genes and subtle at the level of individual genes. Hence, we performed gene set enrichment analysis using the software GSEA [56] and Molecular Signatures Database (MSigDB) [56] to identify whether a set of genes in specific GO terms or pathways terms shows significant differences in Con and Tg groups. Briefly, we input the gene expression matrix and rank genes by the Signal2Noise normalization method. Enrichment scores and *P* value was calculated in default parameters. Gene sets with the parameter of normalized enrichment score (NES) ≥ 1, *P* value < 0.05, and false discovery rate (FDR) < 0.25 were considered significantly enriched.

### Statistical analysis

The data were presented as mean ± standard error of the mean (SEM). For each measured variable in column analyses, the D’Agostino & Pearson normality test was performed to assess whether values were normally distributed. If variables were normally distributed, comparisons between two groups were done by using the unpaired t test, whereas comparisons between multiple groups were conducted by One-Way ANOVA followed by the post hoc Tukey’s multiple comparisons. Values with *P* < 0.05 were considered statistically significant.

## Results

### *T*. *gondii* infection impairs prefrontal cortex-dependent goal-directed behavior in the mice

**Fig 1:**
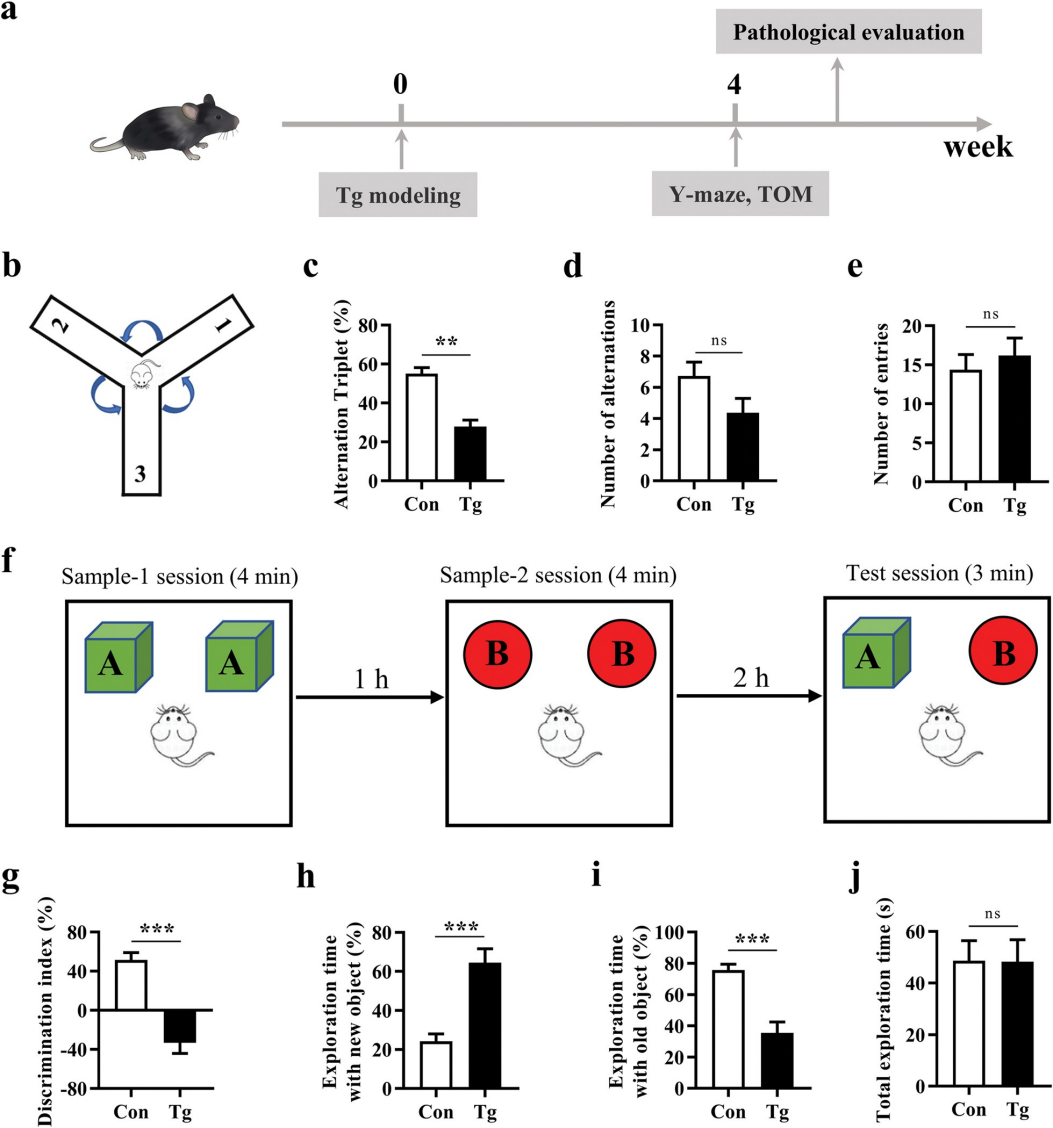
*Toxoplasma gondii* infection impairs prefrontal cortex associated goal-directed behaviors in mice. **a** Schematic timeline for the model establishment of cognitive deficits induced by *T*. *gondii* infection in mice. **b-e** Y-maze test was performed to evaluate the spatial working memory of the mice. **b** Y-maze test schematic diagram. **c** Percentage of alternation tripelet. **d** Number of alternations. **e** Number of entries. **f-j** Temporal order memory (TOM) test was performed to estimate the temporary order memory of the mice. **f** Flow chart of TOM test. **g** Discrimination index. **h** Percentage of time exploring the new object. **i** Percentage of time exploring the old object. **j** Total exploration time of objects in the test phase. n = 11 mice for each group. Con: control group; Tg: *T*. *gondii*-infected group. Values are presented as mean ± SEM. **P < 0.01, ***P < 0.001.

We first investigated whether *T*. *gondii* infection could induce the deficits of goal-directed behavior using Y-maze and TOM tests. The schematic timeline for the behavior tests is shown in Fig 1A. In the Y-maze test, we observed that the infected group had a lower percentage of spontaneous alternations compared with the control group (*P* < 0.01, Fig 1B and 1C), while the number of alternations and the number of entries were not significantly different between the two groups (Fig 1D and 1E). In the TOM test, we found that the discrimination index in the Tg group was significantly lower than that in the control group (*P* < 0.001, Fig 1F and 1G). Detailed speaking, the infected mice spent relatively more time with the new object and less time with the old object compared with control mice (both *P* < 0.001, Fig 1H and 1I). The total exploration time of objects during the test phase was comparable between the two groups (Fig 1J). Taken together, these data suggest that *T*. *gondii* infection induces the deficits of prefrontal cortex-dependent goal-directed behavior.

### *T*. *gondii* infection causes neurites degeneration and the decline of dendritic spine density in the prefrontal cortex of mice

**Fig 2:**
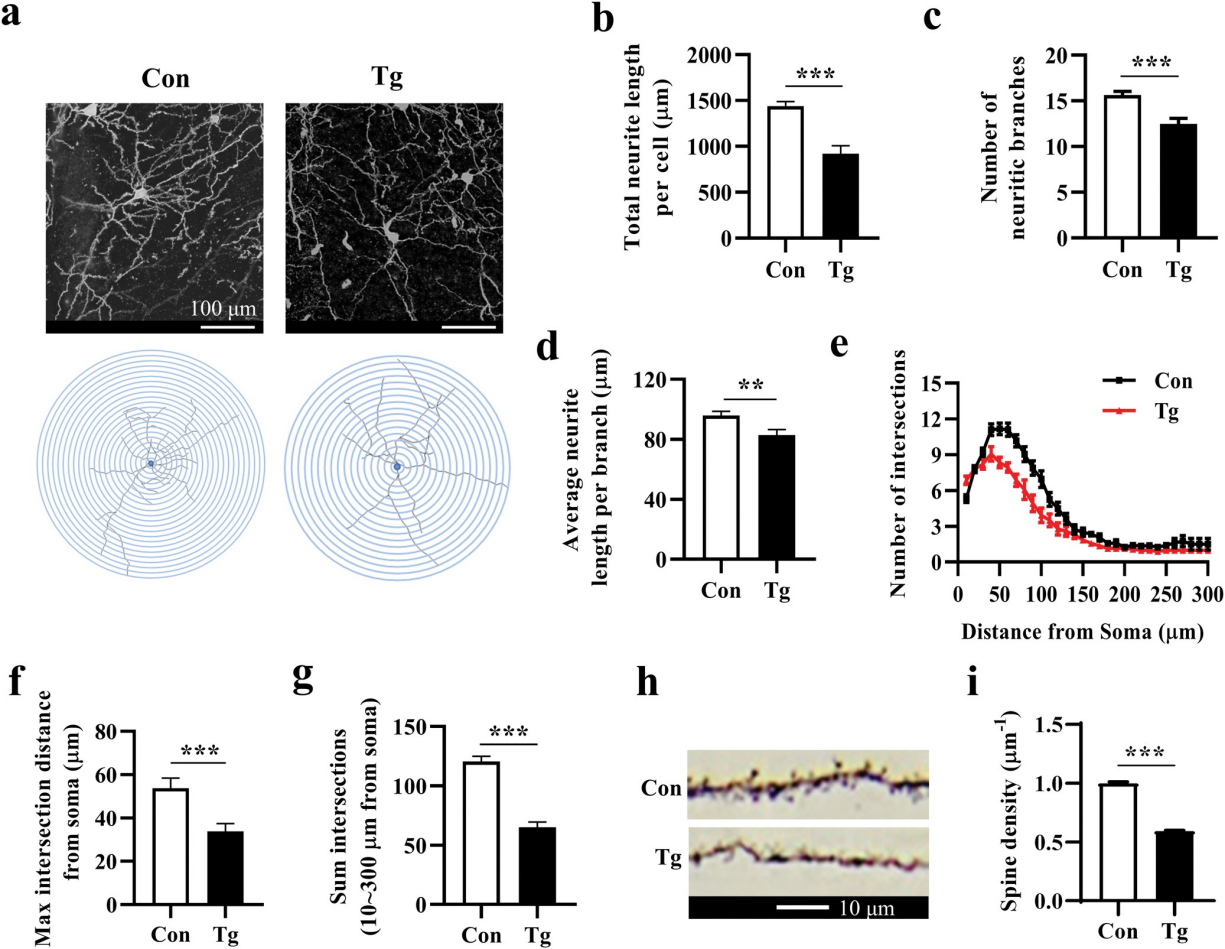
*Toxoplasma gondii* infection causes the neurites degeneration and the decline of dendritic spine density in the prefrontal cortex of mice. **a** Representative images of pyramidal neurons in the prefrontal cortex (scale bar: 100 μm). **b** Average total neurite length and (**c**) the number of neurite branches per cell (n = 19–21 neurons from 3 mice in each group). **d** Average neurite length per branch (n = 220–298 branches from 3 mice). **e-g** Sholl analysis showing the complexity of pyramidal neurons in the prefrontal cortex of Control and *T*. *gondii*-infected mice (n = 19–26 neurons from 3 mice in each group). **h**, **i** Representative images (scale bar: 10 μm) and quantification of dendritic spines of neurons in the prefrontal cortex (n = 50 neurons from 3 mice in each group). Con: control group; Tg: *T*. *gondii*-infected group. Values are presented as the mean ± SEM. **P < 0.01, ***P < 0.001.

Following the observation of impaired cognitive function, we sought to determine whether *T*. *gondii* infection could induce the structural changes of neurons in the prefrontal cortex of mice. Using the Golgi silver staining, we found that compared with the Con group, three crucial neuron morphology indexes including average total neurite length per cell, number of neurite branches, and average neurite length per branch, were decreased in the Tg group (both *P* < 0.001, Fig 2A–2C; *P* < 0.01, Fig 2D). The complexity of neurons was further analyzed using Sholl analysis. We observed the lowered number of intersections in the Tg group (Fig 2E), which implied the complexity of neurons is decreased post infection. In concrete terms, max intersection distance from the soma and sum intersections (10 ~ 300 μm from the soma) were decreased strikingly in the Tg group compared with the Con group (both *P* < 0.001, Fig 2F and 2G). Moreover, a lower spine density was observed in the Tg group (*P* < 0.001, Fig 2H and 2I). Therefore, *T*. *gondii* infection led to the neurite degeneration and the decreased neuronal complexity in the prefrontal cortex of mice.

### *T*. *gondii* infection undermines the synaptic ultrastructure in the prefrontal cortex of mice

**Fig 3:**
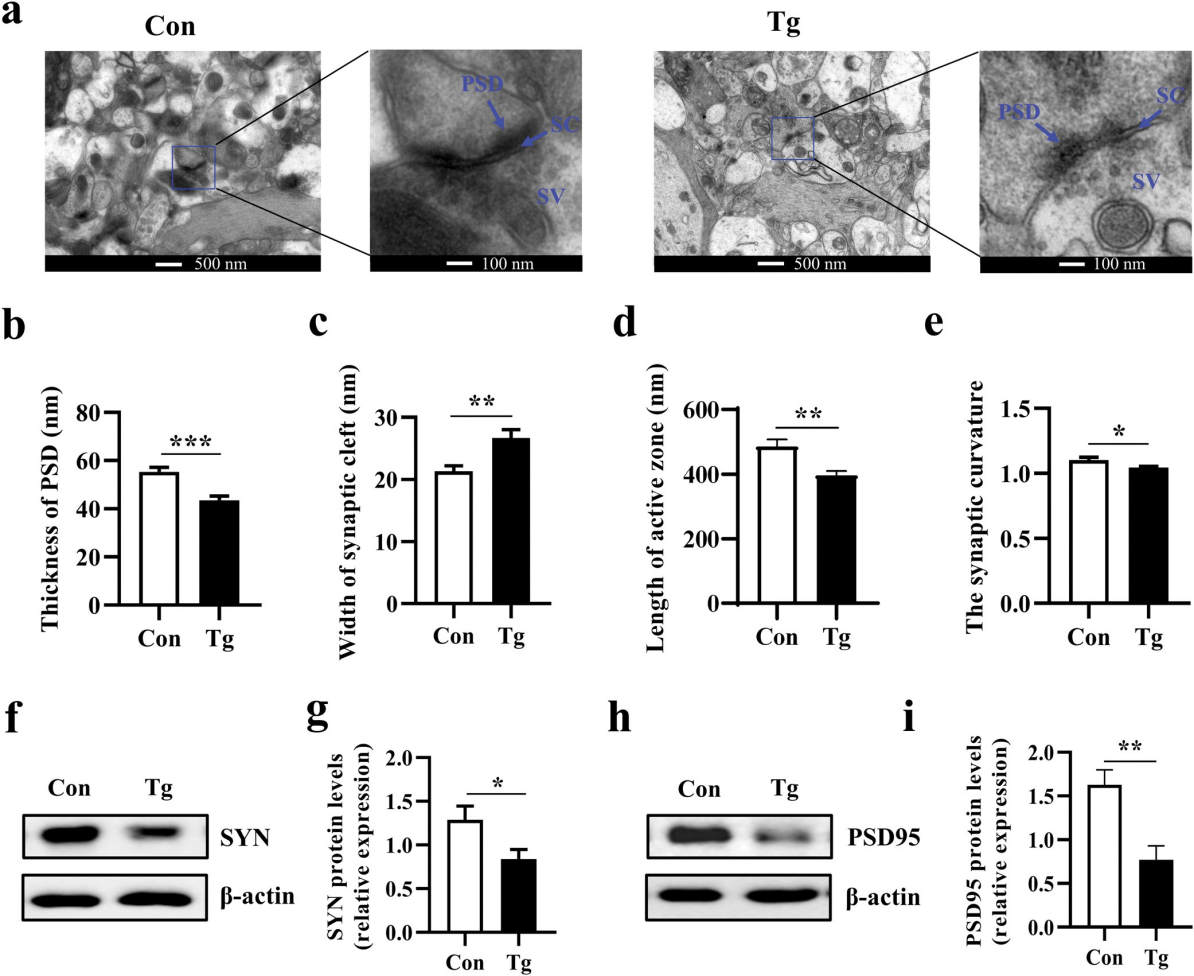
*Toxoplasma gondii* infection undermines the synaptic ultrastructure in the prefrontal cortex of mice. **a** The ultrastructure of synapses in the PFC region of mice on the electron micrograph. The enlarged images on the right side (scale bar: 100 nm) were from the boxed area in the left side images (scale bar: 500 nm). **b-e** Image analysis of the synaptic ultrastructure with software ImageJ (n = 3, 6 images per mouse).**b** Thickness of postsynaptic density. **c** Width of the synaptic cleft. **d** Length of the active zone. **e** The synaptic curvature. **f-i** Representative plots and relative levels of synaptophysin (SYN) and postsynaptic density 95 (PSD95) in the prefrontal cortex of control and *T*. *gondii*-infected mice (n = 5–6). Con: control group; Tg: *T*. *gondii*-infected group; PSD: postsynaptic density; SC: synaptic cleft; SV: synaptic vesicle. Values are presented as mean ± SEM. *P < 0.05, **P < 0.01, ***P < 0.001.

Using transmission electron microscopy, we examined the synapse ultrastructure in the prefrontal cortex of mice. In the Tg group, the thickness of the postsynaptic density was significantly decreased while the width of the synaptic cleft was increased (*P <* 0.001, Fig 3A and 3B; *P* < 0.01, Fig 3C). Moreover, the length of the active zone and the synaptic curvature were decreased in the Tg group (*P <* 0.01, Fig 3D; *P* < 0.05, Fig 3E). In addition, the protein expression of synaptophysin (SYN) and postsynaptic density protein 95 (PSD-95), the pre- and postsynaptic function associated makers, was significantly downregulated in the prefrontal cortex post infection (*P* < 0.05, Fig 3F and 3G; *P* < 0.01, Fig 3H and 3I). These data provide strong evidence that *T*. *gondii* infection impairs the synaptic ultrastructure and neural connectivity in the prefrontal cortex of mice.

### RNA sequencing characterizes the unique profile of cognitive dysfunction in the prefrontal cortex of mice infected by *T*. *gondii*

**Fig 4:**
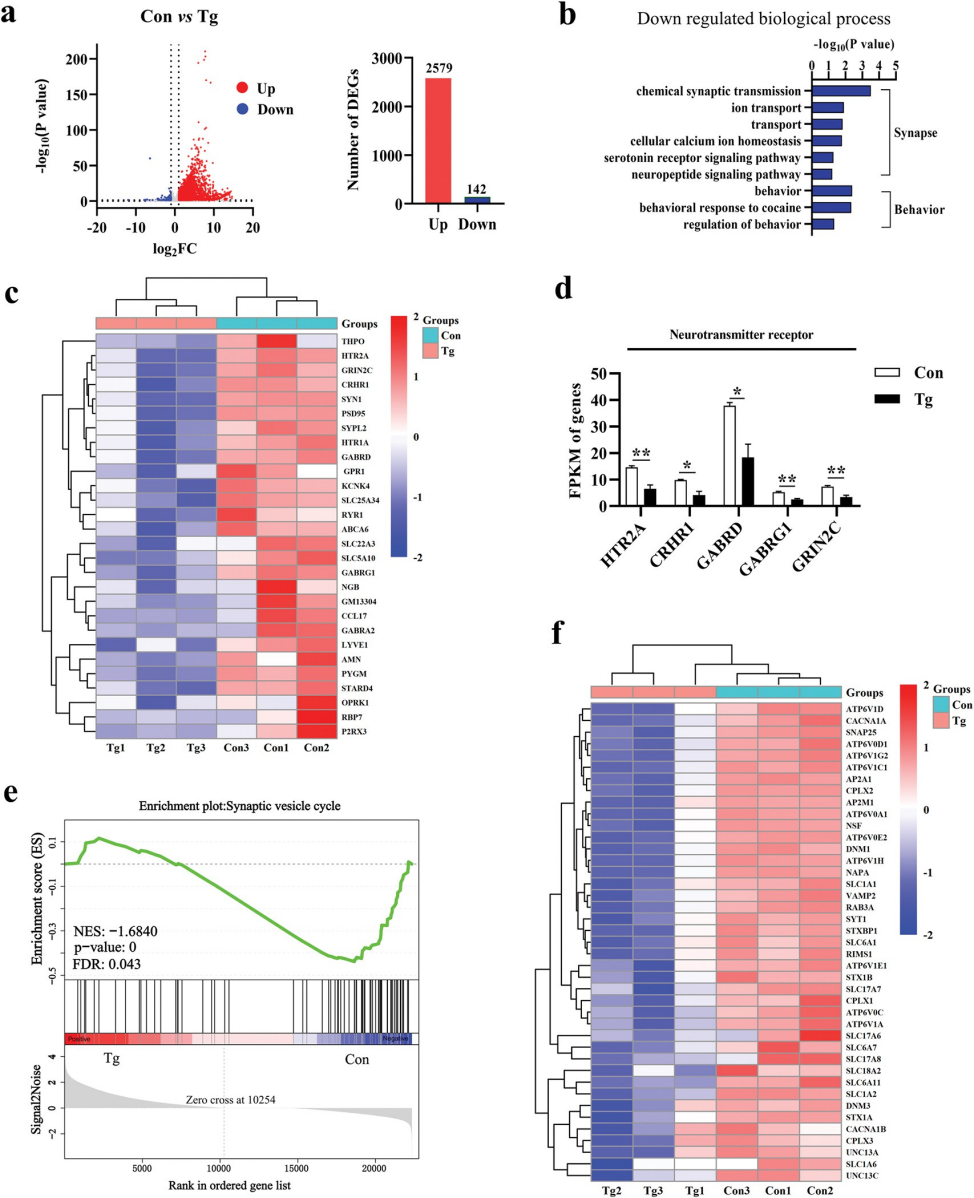
*Toxoplasma gondii* infection impairs the processes of behavior, synaptic plasticity, and transmission in the prefrontal cortex of mice. **a** Volcano plot showing RNA-Seq data of prefrontal cortex from Control and *T*. *gondii*-infected mice. Differentially expressed genes (DEGs) were screened out based on the threshold of *P*-value < 0.05 and fold change (FC) ≥ 2 or ≤ 1/2. The bar chart on the right shows the number of DEGs. **b** Bar chart showing the down-regulated biological processes related to synaptic transmission and behavior. **c** Heatmap depicting the representative genes associated with synaptic plasticity, transmission, and behavior. **d** Normalized expression of selected genes coding neurotransmitter receptor. FPKM (fragment per kilobase of transcript per million mapped reads) value was calculated to quantify gene’s expression abundance and variations, using StringTie software. **e** GSEA analysis revealed that genes involved in the synaptic vesicle cycle were significantly downregulated. **f** Heatmap exhibiting the relative expression of core genes in the enrichment plot e. Con: control group (n = 3); Tg: *T*. *gondii*-infected group (n = 3); NES: normalized enrichment score; FDR: false discovery rate. Values are presented as mean ± SEM. *P < 0.05, **P < 0.01, ***P < 0.001.

To investigate the potential underlying molecular mechanism of the cognitive deficits induced by *T*. *gondii* infection, we evaluated the expression pattern of the prefrontal cortex tissues using RNA sequencing. In compared to the control group, *T*. *gondii* infection caused 2579 upregulated and 142 downregulated differentially expressed genes (DEGs) (Fig 4A). Interestingly, Gene Ontology (GO) analysis showed that the downregulated DEGs were significantly (*P* < 0.05) enriched in 23 biological processes (S2 Table), among which 9 biological processes are related with synapse and behavior (Fig 4B). Moreover, the relative abundance of DEGs that regulate pathways such as “behavioral response to cocaine”, “behavior”, “neuropeptide signaling pathway”, “serotonin receptor signaling pathway”, “cellular calcium ion homeostasis”, “transport,” and “chemical synaptic transmission” became much lower post *T*. *gondii* infection (Fig 4C). Notably, the relative abundance of SYN1 and PSD95, the markers of synapse ultrastructure, were also significantly downregulated post infection (Fig 4C).

Furthermore, we observed that lots of downregulated genes were clustered in neurotransmitter receptors such as HTR2A, CRHR1, GABRD, and GRIN2C (Fig 4D), which are involved in encoding the subunit of 5-hydroxytryptamine, corticotropin-releasing factor, gamma-aminobutyric acid (GABA) and glutamate (NMDA) receptor, respectively. Hence, these data imply the neurotransmitter transmission was abnormal post infection. To assess the function of synaptic transmission in a whole picture, we performed Gene Set Enrichment Analysis (GSEA) to determine the enriched gene sets of specific GO or KEGG pathways. From the cognitive perspective, 4 significantly enriched gene sets (NES ≥ 1, *P* < 0.05, and FDR < 0.25) including “Synaptic vesicle cycle”, “GABA receptor activity”, “Glutamatergic synapse,” and “Dopaminergic synapse” were identified (Figs 4E and S1A–S1C) and the relative abundance of the core enrichment genes in the “Synaptic vesicle cycle” were presented in the heatmap (Fig 4F). We uncovered that the genes regulating the “Synaptic vesicle cycle” were largely downregulated post infection, suggesting the blockage of neurotransmitter release. Collectively, these results indicate that *T*. *gondii* infection disrupts the synaptic transmission accompanied by the dysregulation of the neurotransmitter systems.

### *T*. *gondii* infection induces neuroinflammation in the prefrontal cortex of mice

**Fig 5:**
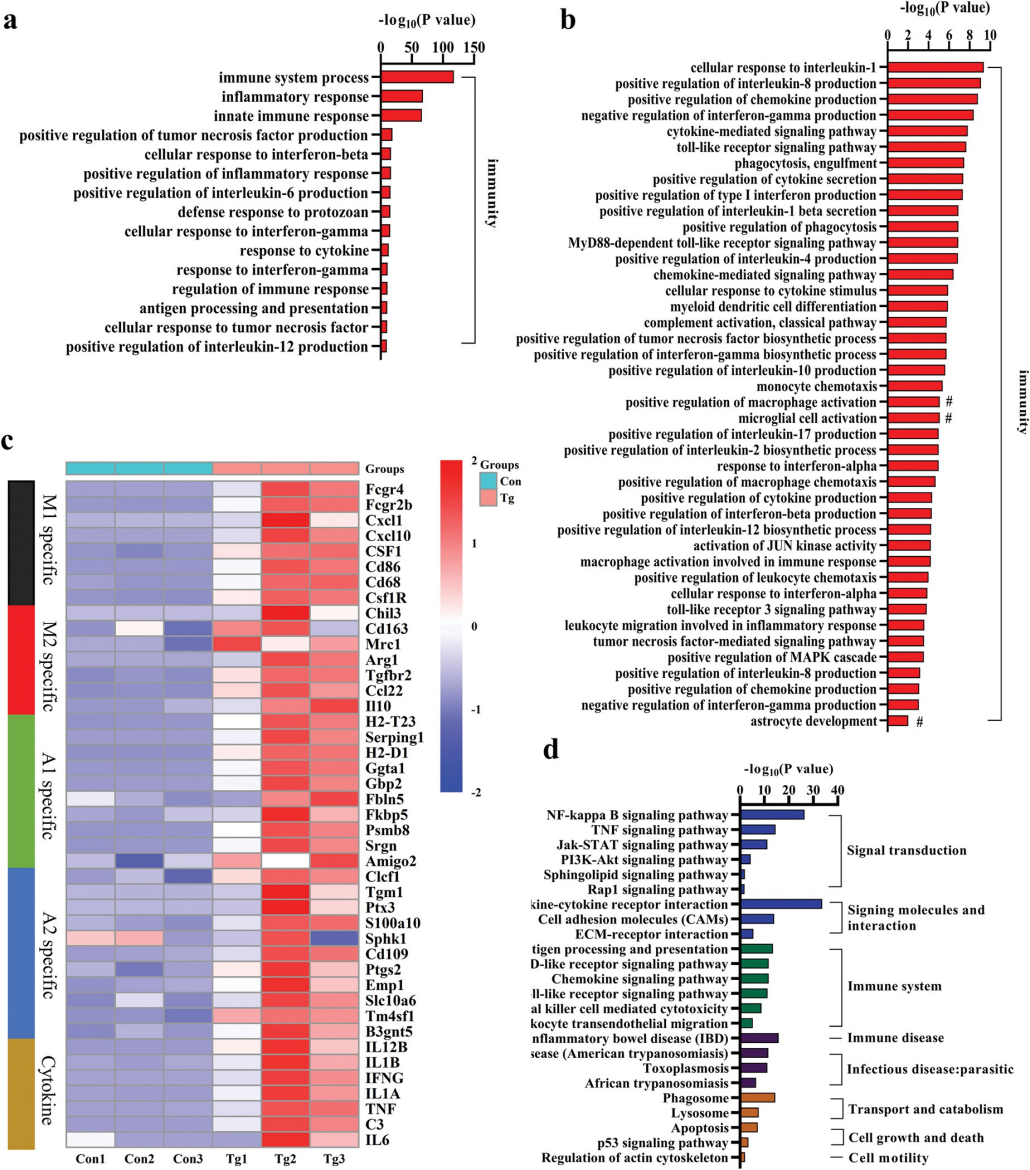
*Toxoplasma gondii* infection triggers extensive neuroinflammation in the prefrontal cortex in mice. **a, b** Bar charts showing the up-regulated biological processes related to immunity. **c** Heatmap depicting the relative expression of representative genes related to cytokine or markers of immune activation. **d** The enriched KEGG pathways. Con: control group (n = 3); Tg: *T*. *gondii*-infected group (n = 3).

RNA-seq of the prefrontal cortex identified that upregulated DEGs were significantly enriched in 672 GO biological processes (S3 Table) and 76 KEGG pathways (S4 Table). We noticed that most enriched biological processes were linked with “inflammation”, “immune response” or “cytokine production” (Fig 5A and 5B). Expectedly, *T*. *gondii* infection significantly upregulated “positive regulation of interleukin-6 production” (Fig 5A). Moreover, other proinflammatory events such as “cellular response to tumor necrosis factor TNF-α”, “positive regulation of Interleukin-12 production,” and “cellular response to Interleukin 1” were also enriched (Fig 5A and 5B). In addition, “microglia cell activation”, “positive regulation of macrophage activation” and “astrocyte development”, were observed in the enriched biological processes. Fig 5C listed the representative activation makers regarding neuroinflammation. The infection led to the upregulation of both the activated markers (CD86, IL1β, and IL6; H2-D1, Ggta1, Fbln5, and Psmb8) associated with M1 microglia and A1 astrocytes [24, 57]. Furthermore, KEGG analysis showed that the top 2 enriched pathways were “Cytokine-cytokine receptor interaction” and “NF-kappa B signaling pathway” (Fig 5D). These results support that *T*. *gondii* infection triggers extensive neuroinflammation, thereby damaging the equilibrium in the prefrontal cortex of mice.

### *T*. *gondii* infection modulates the metabolic reprogramming in the prefrontal cortex of mice

**Fig 6:**
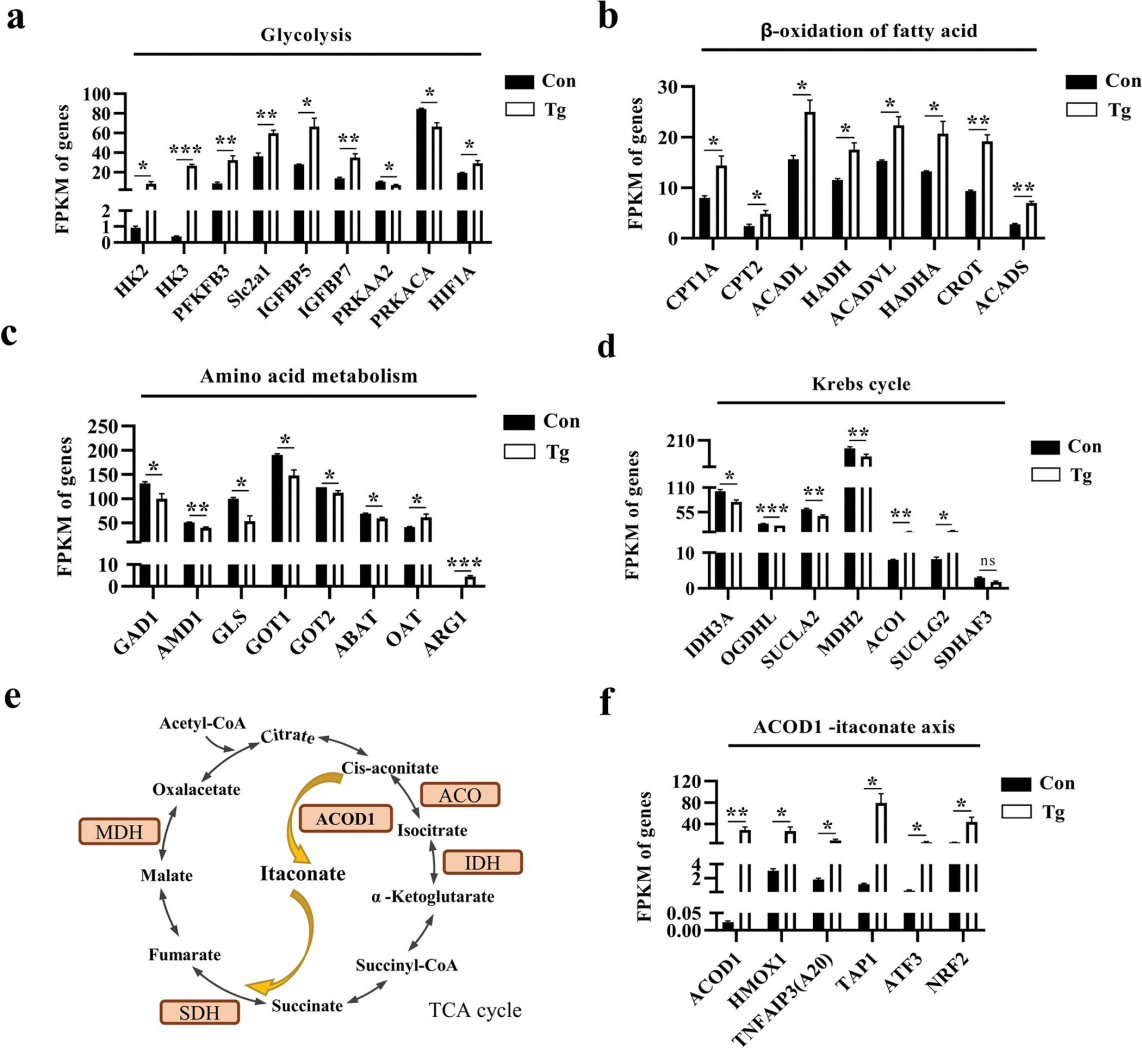
*Toxoplasma gondii* infection modulates metabolic reprogramming in the prefrontal cortex of mice. Normalized expression of selected genes regulating glycolysis (**a**), fatty acid β-oxidation (**b**), amino acid metabolism (**c**), Krebs cycle (**d**), and ACOD1-itaconate axis (**f**). **e** The process of the Krebs cycle. Con: control group (n = 3); Tg: *T*. *gondii*-infected group (n = 3). FPKM (fragment per kilobase of transcript per million mapped reads) value was calculated to quantify gene’s expression abundance and variations, using StringTie software. Values are presented as mean ± SEM. *P < 0.05, **P < 0.01, ***P <0.001.

Using RNA-seq, we observed glycolysis, the typical phenotype of pro-inflammatory response, was reinforced (Fig 6A), which was reflected by the dramatically up-regulated expression of key genes (HK2, HK3, and PFKFB3) coding rate-limiting enzymes in the pathway. In parallel, positive regulators, such as HIF-1α, a master transcriptional regulator of glycolysis [58], and IGFBP5, capable of activating IGF1R-AKT to increase glycolysis [59], were significantly upregulated post infection (Fig 6A). On the contrary, acting as the negative regulators, PRKAA2 and PRKACA, encoding the subunit of AMP-activated protein kinase (AMPK), which have been proven to inhibit glycolysis via AMPK-mTOR-HIF-1α pathway [27], were downregulated post infection (Fig 6A). Concomitantly, *T*. *gondii* infection affected the gene expression related to fatty acid β-oxidation and amino acid metabolism (Fig 6B and 6C).

Intriguingly, as a part of glucose metabolism, the Krebs cycle in mitochondria seemed to be impaired by *T*. *gondii* infection (Fig 6D). The relative abundance of the genes (IDH3A and OGDHL) encoding the subunit of isocitrate dehydrogenase (IDH) and α-ketoglutarate dehydrogenase complex (α-KGDHC) respectively, two rate-limiting enzymes in Krebs cycle, was significantly downregulated in *T*. *gondii*-infected group. This change is speculated to block the Krebs cycle. Moreover, a decreased expression of enzyme subunits (MDH2 and SUCLA2) and an elevated expression of ACO1 and SUCLG2 in the cycle were observed post infection. Interestingly, the ACOD1-itaconate axis, previously proven to be a vital node that controls the immunity and metabolism in macrophages [28], was disordered in the prefrontal cortex post *T*. *gondii* infection. ACOD1 encoding cis-aconite decarboxylase can catalyze the conversion of cis-aconitate to itaconate (Fig 6E). RNA-seq showed that the expression of ACOD1 and its downstream genes (HMOX1, TNFAIP3, TAP1, ATF3, and NRF2) [60] was significantly upregulated post infection (Fig 6F). Taken together, these results indicate that *T*. *gondii* infection induces metabolic reprogramming in the prefrontal cortex of the infected mice.

### Dimethyl itaconate suppresses the activation of microglia and astrocytes and neuroinflammation in the PFC of *T*. *gondii*-infected mice

**Fig 7:**
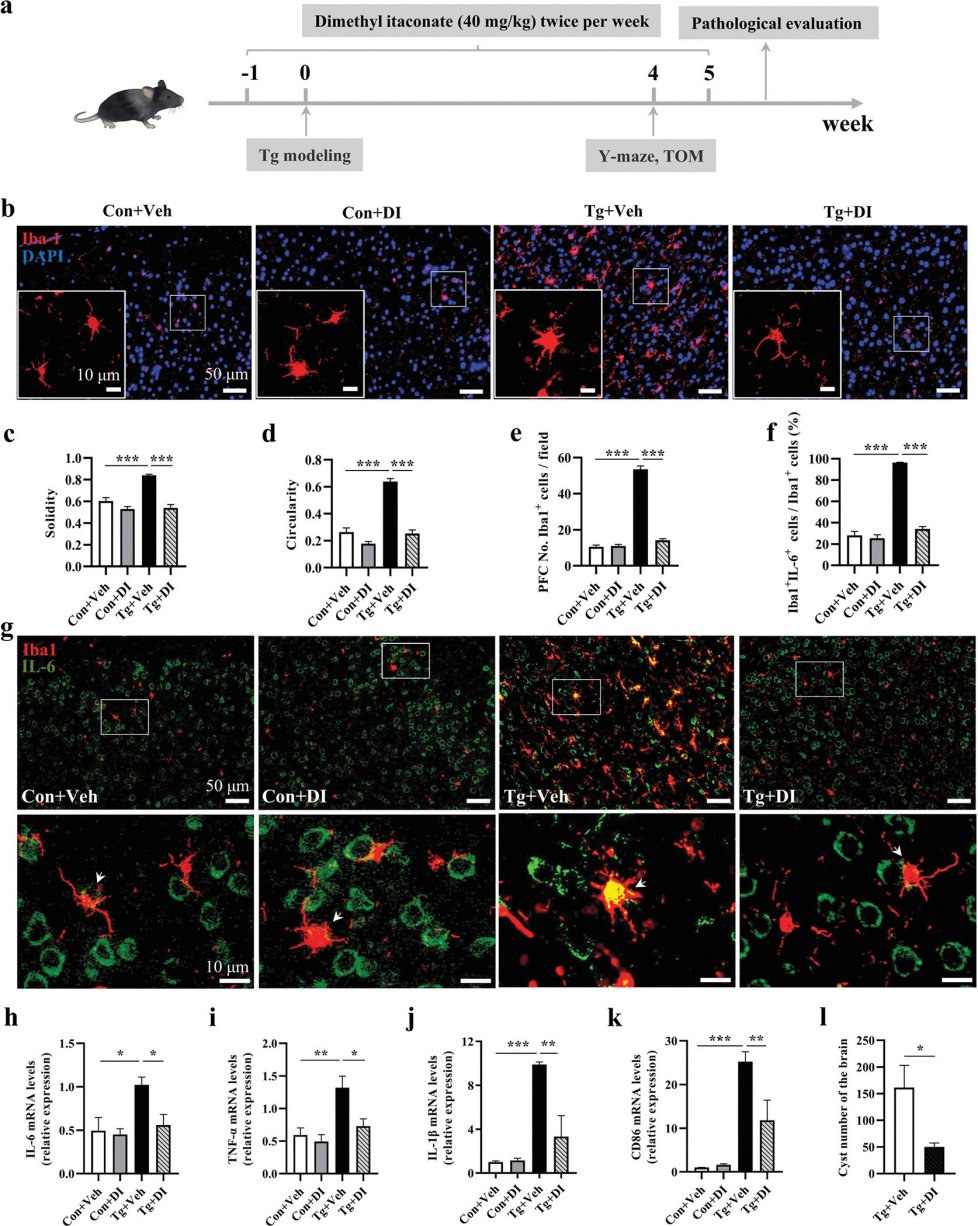
Dimethyl itaconate suppresses the activation of microglia and neuroinflammation in the prefrontal cortex of *T*. *gondii*-infected mice. **a** Schematic timeline for DI supplementation to prevent cognitive deficits induced by *T*. *gondii* infection in mice**. b** Representative immunofluorescent staining of the Iba1^+^ cells in the PFC region of mice (scale bar: 50 μm). The enlarged image captured from the box was marked with a solid line (scale bar: 10 μm). Characterization of microglia morphology via cell solidity (**c**) and circularity (**d**) (n = 21 cells per group). Resting microglia are highly ramified while activated microglia present an amoeboid shape, with no or small ramifications. Activated microglia are characterized by a higher circularity and solidity. **e** Quantification of Iba1^+^ microglia in the PFC (n = 3, 5 images per mouse) **f** Percentage of Iba1^+^IL-6^+^ cells in Iba1^+^ cells (n = 3, 5 images per mouse). **g** Double immunofluorescence staining for Iba1 (red) and IL-6 (green) in PFC, white arrows represent Iba1^+^IL-6^+^ cells (scale bar: 50 μm or 10 μm). **h-k** mRNA expression of IL-6, TNF-α, IL-1β and CD86 in the PFC (n = 6). **l** Cyst enumeration in the brain from Tg+Veh and Tg+DI group mice (n = 6). DAPI: nuclear staining; Iba1: ionized calcium-binding adapter molecule 1; PFC: prefrontal cortex. Con+Veh: control mice with Vehicle control treatment; Con+DI: control mice with DI treatment; Tg+Veh: *T*. *gondii* infected mice with Vehicle control treatment; Tg+DI: *T*. *gondii* infected mice with DI treatment. Values are presented as mean ± SEM. *P < 0.05, **P < 0.01, ***P < 0.001.

Observing the activated ACOD1-itaconate axis post infection, we were interested in whether the administration of Dimethyl itaconate (DI), a cell-permeable itaconate derivative, could modulate the neuroinflammation induced by *T*. *gondii*. The intervention strategy of DI was shown in Fig 7A. Using immunofluorescent labeling of Iba1, we firstly investigated the morphology of microglia in the prefrontal cortex (Fig 7B). In comparison to the Con+Veh group, the majority of microglia of Tg+Veh mice showed the activated phenotype with the enlarged soma, the higher solidity and the circularity (both *P* < 0.001, Fig 7B–7D). Moreover, the number of Iba1^+^ microglia in the Tg+Veh group was significantly increased compared to the Con+Veh group (*P* < 0.001, Fig 7E). While DI supplementation significantly inhibited the morphological transformation and decreased the number of Iba1+ microglia in infected mice (all *P* < 0.001, Fig 7B–7E). However, DI administration seemed to have a minor effect on these indexes mentioned above in the control mice. Moreover, we assessed the expression profile of inflammatory cytokine IL-6 in microglia. In the Tg+Veh group, the percentages of Iba1^+^IL-6^+^ cells in Iba1^+^ cells were significantly higher than those in Con+Veh and Tg+DI groups (*P* < 0.001, Fig 7F and 7G). Correspondingly, the mRNA expression of IL-6, TNF-ɑ, IL-1β, and CD86 was significantly upregulated post *T*. *gondii* infection, while this upregulation was inhibited after DI preventive treatment (*P* < 0.05, Fig 7H and 7I; *P* < 0.01, Fig 7J and 7K). In addition, the increased number of GFAP^+^ cells was observed in the Tg+Veh group when compared to the Con+Veh group, while DI supplementation strikingly decreased the GFAP^+^ cell number in *T*. *gondii* infected mice (S2A–S2C Fig). Furthermore, we evaluated the effects of DI administration on the burden of *T*. *gondii* cysts in the brain of infected mice. As shown in Fig 7L, the cyst number was significantly decreased in the Tg+DI group compared with the Tg+Veh group (*P* < 0.05). These findings indicate that DI suppressed the neuroinflammation induced by *T*. *gondii* infection.

### Dimethyl itaconate prevents the deficits of goal-directed behavior induced by *T*. *gondii* infection in mice

**Fig 8:**
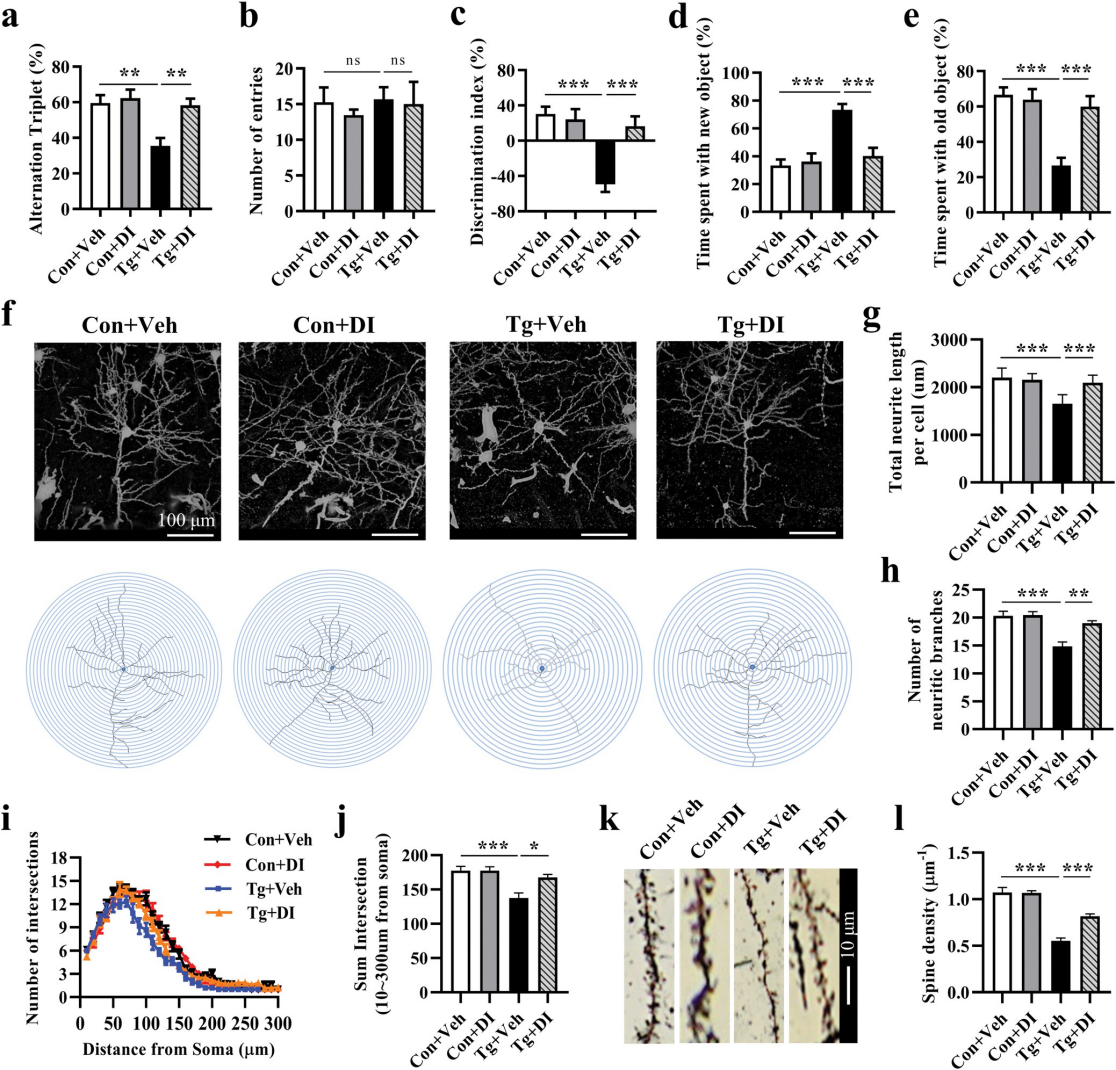
Dimethyl itaconate supplementation prevents the deficits of goal-directed cognitive behavior and neurite degeneration induced by *T*. *gondii*. **a-d** The Y-maze and TOM test were performed to evaluate the protective effect of DI on spatial working memory and temporal order memory of the mice post infection (n = 11). **a** Percentage of alternation triplet. **b** Number of entries. **c** Discrimination index. **d** Percentage of time exploring the new object. **e** Percentage of time exploring the old object. **f** Representative images of pyramidal neurons in PFC (scale bars: 100 μm). **g** Average total neurite length and (**h)** the number of neurite branches per cell (n = 15–21 neurons from 3 mice in each group). **i, j** Sholl analysis showing the complexity of neurons in PFC (n = 15–18 neurons from 3 mice in each group). **k, l** Representative images (scale bar: 10 μm) and quantification of dendritic spines of neurons in the PFC (n = 20–24 neurons from 3 mice in each group). Con+Veh: control mice with Vehicle control treatment; Con+DI: control mice with DI treatment; Tg+Veh: *T*. *gondii* infected mice with Vehicle control treatment; Tg+DI: *T*. *gondii* infected mice with DI treatment. Values are presented as mean ± SEM. *P < 0.05, **P < 0.01, ***P < 0.001.

After determining the anti-inflammatory effect of DI, we furthermore assessed its protective effect on cognition. In the Y-maze test, we found that mice in the Tg+DI group showed an increase in the percentage of alternation triplet compared with the Tg group (*P <* 0.01, Fig 8A). The number of entries was scarcely different between the two groups (Fig 8B). In the TOM test, we observed that DI significantly ameliorated the lowered discrimination index in the infected mice (*P <* 0.001, Fig 8C). Meanwhile, mice in the Tg+DI group relatively spent less time with new object and more time with old object compared with Tg+Veh group (both *P <* 0.001, Fig 8D and 8E). Overall, these results show that DI could prevent the impairment of goal-directed behavior in *T*. *gondii*-infected mice.

### Dimethyl itaconate mitigates neurites degeneration, increases dendritic spines, and improves synaptic ultrastructure in the prefrontal cortex of *T*. *gondii*-infected mice

**Fig 9:**
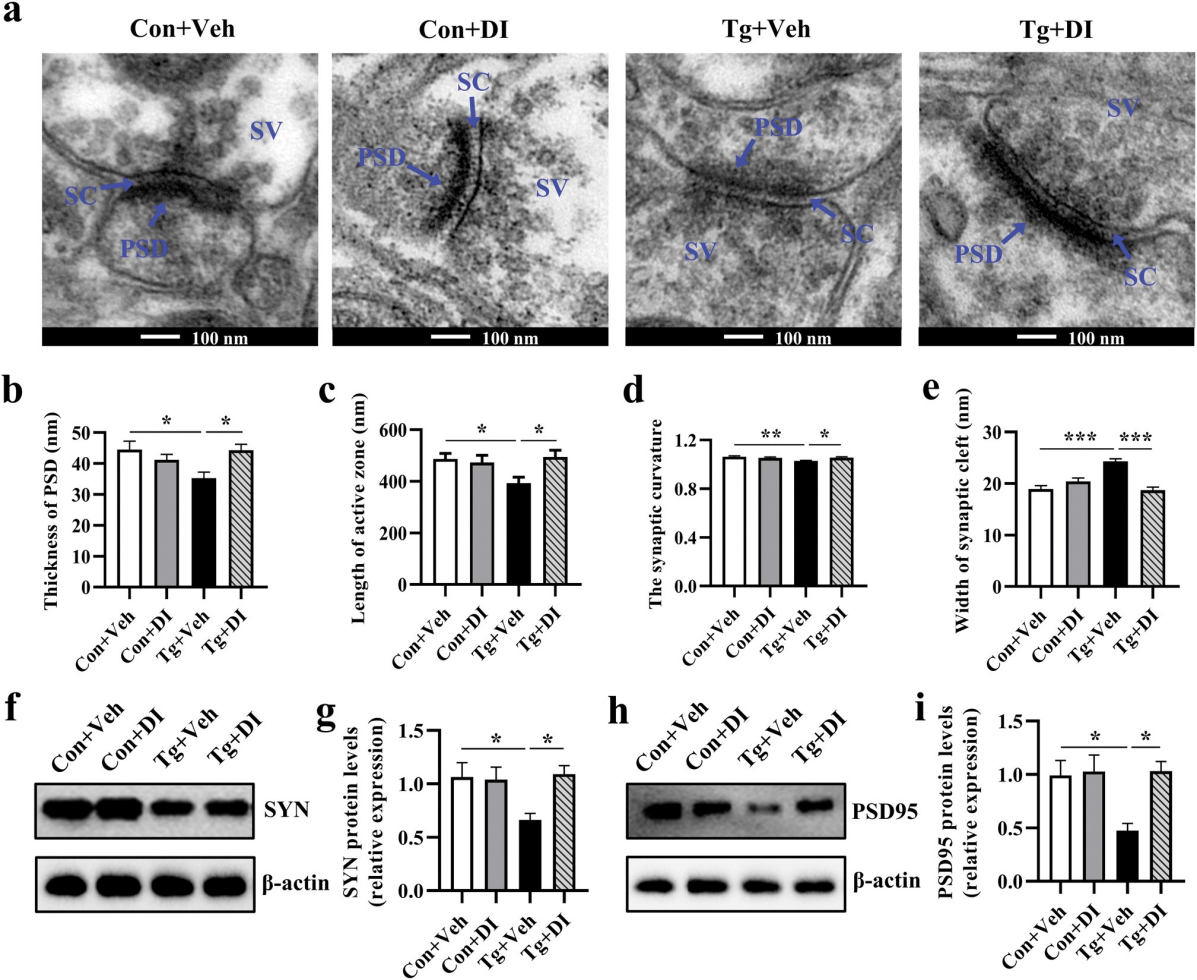
Dimethyl itaconate supplementation improves the impairment of the synaptic ultrastructure in the prefrontal cortex of *T*. *gondii*-infected mice. **a** The ultrastructure of synapses in the PFC region of mice on the electron micrograph (scale bars: 100 nm). **b-e** Image analysis of the thickness PSD, length of the active zone, synaptic curvature and width of synaptic cleft (n = 3, 6 images per mouse). **f-i** The representative bands and relative expression of SYN and PSD95 in the prefrontal cortex of mice (n = 8). PSD: postsynaptic density; SC: synaptic cleft; SV: synaptic vesicle; AZ: active zone. Con+Veh: control mice with Vehicle control treatment; Con+DI: control mice with DI treatment; Tg+Veh: *T*. *gondii* infected mice with Vehicle control treatment; Tg+DI: *T*. *gondii* infected mice with DI treatment. Values are presented as mean ± SEM. *P < 0.05, **P < 0.01, ***P <0.001.

Golgi staining showed that the DI supplementation increased the total neurite length per cell (*P* < 0.001, Fig 8F and 8G) and the number of neurite branches (*P* < 0.01, Fig 8H). Additionally, Sholl analysis showed that DI mitigated the lowered number of neuronal intersections in prefrontal cortex induced by *T*. *gondii* (*P* < 0.01, Fig 8I and 8J), indicating the improvement of neuronal complexity. In parallel, the spine density in Tg+DI group mice was significantly increased compared with the Tg+Veh group (*P* < 0.001, Fig 8K and 8L). Transmission electron microscopy showed that compared with the Tg+Veh group, DI increased the thickness of the postsynaptic density, elongated the length of the active zone, elevated the synaptic curvature, and shortened the synaptic cleft in *T*. *gondii*-infected mice (all *P* < 0.05, Fig 9A-d; *P* < 0.001, Fig 9E). In line with the improvement of synaptic ultrastructure, DI supplementation upregulated the protein expression of SYN and PSD95, two important markers of synaptic function, in *T*. *gondii* infected mice (both *P* < 0.05, Fig 9F–9I). In summary, these results demonstrate the beneficial effect of DI supplementation on neuronal complexity and synaptic ultrastructure, and this could be proposed as an explanation for improving the cognitive deficits induced by *T*. *gondii* infection.

### Dimethyl itaconate has a therapeutic effect on *T*. *gondii-*induced deficits of goal-directed behavior

**Fig 10:**
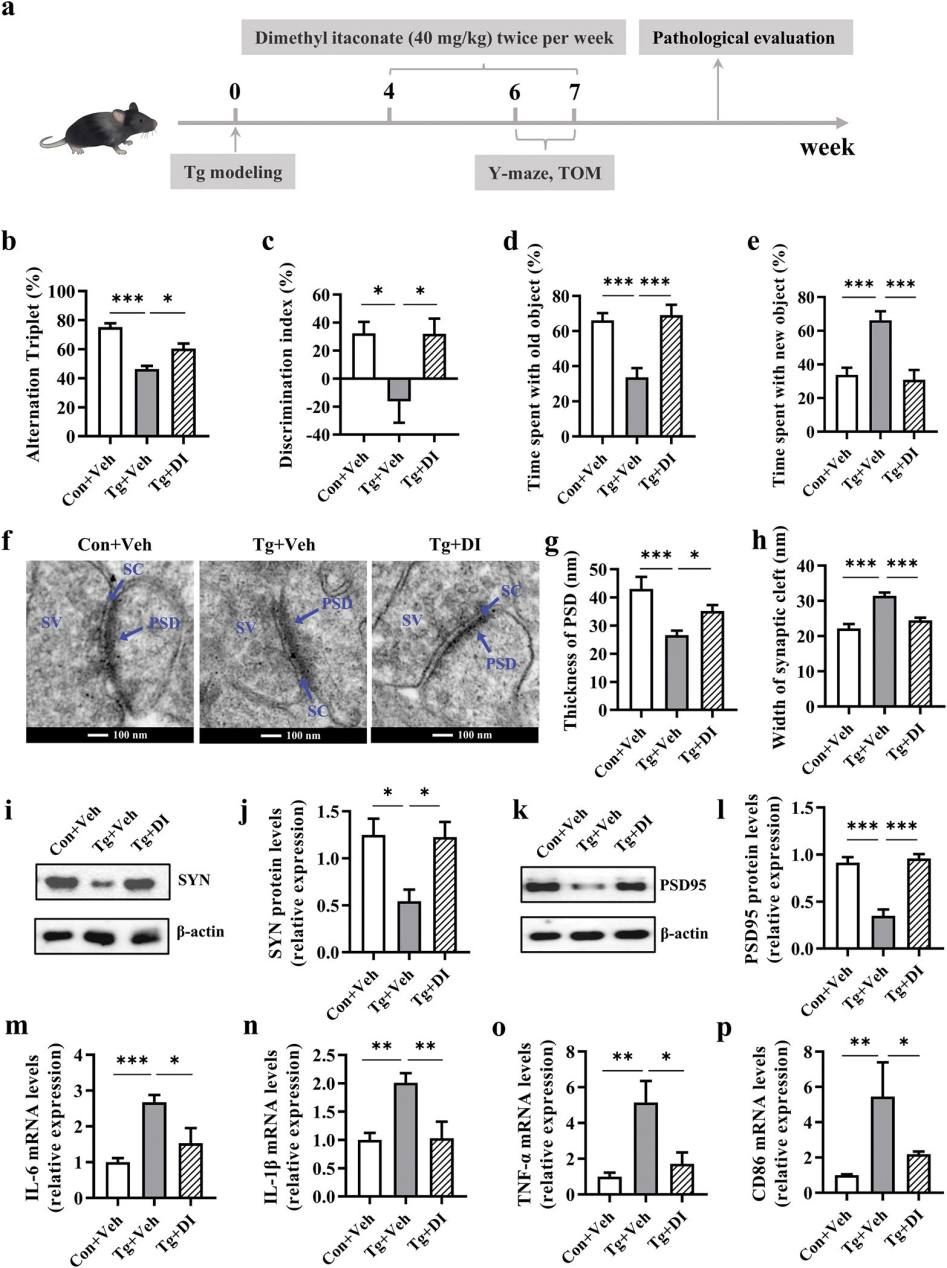
Dimethyl itaconate has a therapeutic effect on *T*. *gondii-*induced deficits of goal-directed behavior. **a** Schematic timeline for DI treatment on cognitive deficits induced by *T*. *gondii* infection in mice. **b** The percentage of alternation triplet (n = 11). **c** The discrimination index (n = 11). **d** Time spent with the old object. **e** Time spent with the new object. **f** The ultrastructure of synapses in the PFC region of mice on the electron micrograph **(**scale bars: 100 nm). **g, h** Image analysis of the thickness of PSD and width of synaptic cleft (n = 3, 6 images per mouse). **i-l** The representative bands and relative expression of SYN and PSD95 in the prefrontal cortex of mice (n = 6). **m-p** mRNA expression of IL-6, IL-1β, TNF-α and CD86 in prefrontal cortex (n = 3–6). PSD: postsynaptic density; SC: synaptic cleft; SV: synaptic vesicle. Con+Veh: control mice with Vehicle control treatment; Tg+Veh: *T*. *gondii* infected mice with Vehicle control treatment; Tg+DI: *T*. *gondii* infected mice with DI treatment. Values are presented as mean ± SEM. *P < 0.05, **P < 0.01, ***P <0.001.

To further explore whether DI possesses a therapeutic effect, we administrated DI to *T*. *gondii*-infected mice already showing the deficits of goal-directed behavior. The strategy was shown in Fig 10A. In the Y-maze test, DI treatment significantly increased the percentage of alternation triplet in infected mice (*P* < 0.05, Fig 10B). In the TOM test, mice with DI treatment showed an elevated discrimination index, spending more time with the old object and less time with the novel object compared with the untreated Tg mice (*P* < 0.05, Fig 10C; both *P* < 0.001, Fig 10D and 10E). Moreover, the Tg+DI group exhibited thicker postsynaptic densities and narrower synaptic cleft in the prefrontal cortex of infected mice (*P* < 0.05, Fig 10F and 10G; *P* < 0.001, Fig 10H). Correspondingly, DI upregulated the mRNA and protein expression of SYN and PSD95 in the *T*. *gondii* infected mice (*P* < 0.05, S3A and S3B Fig; *P* < 0.05, Fig 10I and 10J; *P* < 0.001, Fig 10K and 10L). In addition, DI suppressed the mRNA expression of pro-inflammatory molecules including IL-6, IL-1β, TNF-ɑ, and CD86 (all *P* < 0.05, Fig 10M, 10o and 10p; *P* < 0.01, Fig 10N). Furthermore, DI treatment significantly decreased the number of the cysts in the brains of infected mice (*P* < 0.05, S3C Fig). Collectively, the results demonstrate that DI could treat the deficits of the goal-directed behavior induced by *T*. *gondii* by suppressing the neuroinflammation and alleviating the impairment of synaptic ultrastructure.

## Discussion

In the present study, we demonstrated that *T*. *gondii* infection undermined prefrontal cortex-dependent goal-directed behavior, which was accompanied by degenerated neurites, decreased density of dendritic spines and impaired synaptic ultrastructure. Moreover, the infection significantly inhibited the expression of key genes associated with synapse plasticity, transmission, and behavior; however, the infection robustly upregulated neuroinflammation, characterized by the activation of microglia and astrocytes. In addition, the enhanced glycolysis and fatty acid β-oxidation, and blockage of the Krebs cycle, were identified post infection based on the relative abundance of the genes coding the key enzymes in those pathways. Interestingly, the ACOD1-itaconate axis was disordered post *T*. *gondii* infection. We reported that the administration of DI, a derivative of itaconate, could prevent and treat the deficits of the goal-directed behavior induced by the parasite via alleviating neuroinflammation and improving the impairment of neuron integrity and synaptic ultrastructure.

Synaptic plasticity, regulating the neuronal circuits, has long been considered an irreplaceable part of cognition [61]. Observing the impaired working memory and temporal order memory in *T*. *gondii*-infected mice, we further identified a striking neuropathologic lesion and undermined synaptic ultrastructure, suggesting that infection impaired neural circuits and synaptic transmission in the prefrontal cortex. Previous studies similarly uncovered the reduction of spine density [62] and modified synapse connectivity [63] in mice with chronic toxoplasmosis. These results indicate that the altered neural circuits and synaptic ultrastructure are the neuropathological foundation of *T*. *gondii*-induced cognitive impairment. Moreover, we showed that the biological process of synaptic vesicle cycling was disrupted post *T*. *gondii* infection. Dynamic synaptic vesicle recycling is critical for maintaining normal synaptic transmission. In the model of schizophrenia, working memory impairment in mice is considered to be a consequence of altered synaptic vesicle cycling [64]. To determine this alternation, we examined the expression of synapse-related proteins like SYN and PSD95. We found the two proteins were significantly downregulated in the infected mice. Located in presynaptic terminals and synaptic vesicles, SYN participates in vesicle clustering, neurotransmitter release, and neuroplasticity, which is crucial for intact synaptic function [65, 66]. However, deletion of the Syn genes could induce epilepsy and symptoms of autism spectrum disorders (ASD) in mice [67]. Thus, it can be speculated that the deficit of SYN might be a vital node in *T*. *gondii*-induced cognitive impairment. Additionally, our GSEA analysis mapped the enrichment plot of the GABAergic and glutamatergic systems. Several studies have reported GABA and glutamate transmission are disordered in the mice brain post *T*. *gondii* infection [62, 68]. Besides, in a large-scale proteomic study, the synaptic proteins, such as glutamate NMDA receptor subunits, were decreased in the brain of *T*. *gondii* infected mice [12], which was consistent with our transcriptomic results. Collectively, these results indicate that *T*. *gondii* infection undermines normal synaptic transmission manifesting as impaired neuronal integrity and synaptic ultrastructure, which may be one of the underlying mechanisms for *T*. *gondii*-associated cognitive deficits.

It is established that neuroinflammation characterized by microglia and astrocyte activation can exert direct detrimental effects on memory, neural plasticity, and neurogenesis [14, 24]. Our results showed that *T*. *gondii* infection robustly increased the number of Iba1^+^ microglia and GFAP^+^ astrocytes and elevated pro-inflammatory cytokine (IL-6, IL-1β, and TNF-α), suggesting massive neuroinflammation was triggered by the parasite. We noticed that the specific markers of A1 astrocyte (*e*.*g*., H2-T23, Serping1, H2-T23) were upregulated post *T*. *gondii* infection. In the physiological state, the astrocytes fulfill a range of homeostatic maintenance functions [69]; whereas, under the inflammatory circumstance, they polarized into the neurotoxic A1 subtype [24]. A previous study has reported that *T*. *gondii* can induce C3-expressing A1 astrocytes via the NF-κB pathway [70]. Our results further confirmed this finding at the transcriptional level. Activated A1 astrocytes kill cortical neurons via secreting a soluble toxin and have been implicated in Alzheimer’s disease (AD), Parkinson and multiple sclerosis [24]. From this perspective, A1 astrocyte is potentially one of the important mediators for neurodegeneration induced by *T*. *gondii*. However, in our double immunofluorescent staining, the percentage of IL-6^+^GFAP^+^ cell was lower than IL-6^+^Iba1^+^ cell post-infection, which indicate that the pro-inflammatory cytokine, such as IL-6, was mainly derived from microglia. As the primary immune cells in the brain, sustained activation of microglia is considered an important mechanism in the progression of many neurodegenerative diseases including multiple sclerosis, stroke, AD, and Parkinson [25]. The inflammatory microglia not only produce a barrage of neurotoxic factors but amplify the neuroinflammation via initiating activation of astrocytes [24], consequently causing neuronal injury and cognitive impairment. In the present study, we identified the upregulated expression of several M1 markers of microglia (IL-6, IL-1β, TNF-ɑ, and CD86) [57], which indicated that *T*. *gondii* infection promotes the polarization of microglia to M1-like phenotype. A previous study report that, besides magnifying inflammation, microglia is capable of phagocyting synapse components via complement-dependent pathways in the early of the disease [71]. Correspondingly, we noticed that the infection upregulated transcripts of members in the complement family, which was in line with the impaired synaptic ultrastructure in the infected mice. Thus, it is very likely that activated microglia and neurotoxic astrocytes in the inflammation milieu caused neuronal injure and synaptic loss, eventually leading to cognitive impairment induced by *T*. *gondii*.

As a novel discipline, Immunometabolism has uncovered that metabolic reprogramming is the basis for immune cell differentiation and function, thereby affecting health and disease [21]. Using RNA sequencing, we found that glycolysis is a primary metabolic phenotype in the prefrontal cortex post infection, because the relative abundance of genes encoding the enzymes in the glycolysis was significantly upregulated. Correspondingly, we observed the extensive upregulation of genes related to inflammation. In a mice model of AD, Aβ-induced microglia inflammation is proved to be dependent on the mTOR-HIF-1α pathway, which is a master regulator of glycolysis [27, 58]. It is therefore speculated that the enhanced glycolysis is associated with upregulated pro-inflammatory response in the brain post *T*. *gondii* infection. Emerging studies have shown that several hallmark outputs of microglia during neuroinflammation, such as cytokine release and nitric oxide (NO) production, are dependent on a metabolic shift characterized by hyperglycolysis [72, 73]. In the present study, we identified that the transcripts of glycolysis-regulatory genes (*e*.*g*., HK2, HK3, HIF1A.) were robustly upregulated, which was consistent with upregulated neuroinflammation in the brain area. On the other hand, we uncovered that the relative abundance of CPT1A and CPT2, which encode Carnitine O-palmitoyltransferase, a rate-limiting enzyme involved in the fatty acid oxidation (FAO), were significantly up-regulated post infection, indicating accelerated FAO pathway. It is reported that the activated FAO contributes to the anti-inflammatory response in microglia [73]. A straightforward explanation is that the mechanism of feedback upregulated the anti-inflammatory response, which was consistent with the upregulated interleukin-10 (IL-10), an anti-inflammatory factor, post infection. Collectively, these data suggest that *T*. *gondii* infection induces subtle metabolic remodeling events in the prefrontal cortex, which triggered the inflammatory response and thereby exerted detrimental effects on cognition function. Hence, targeting these metabolic pathways for therapeutic intervention is proposed as a promising approach.

**Fig 11:**
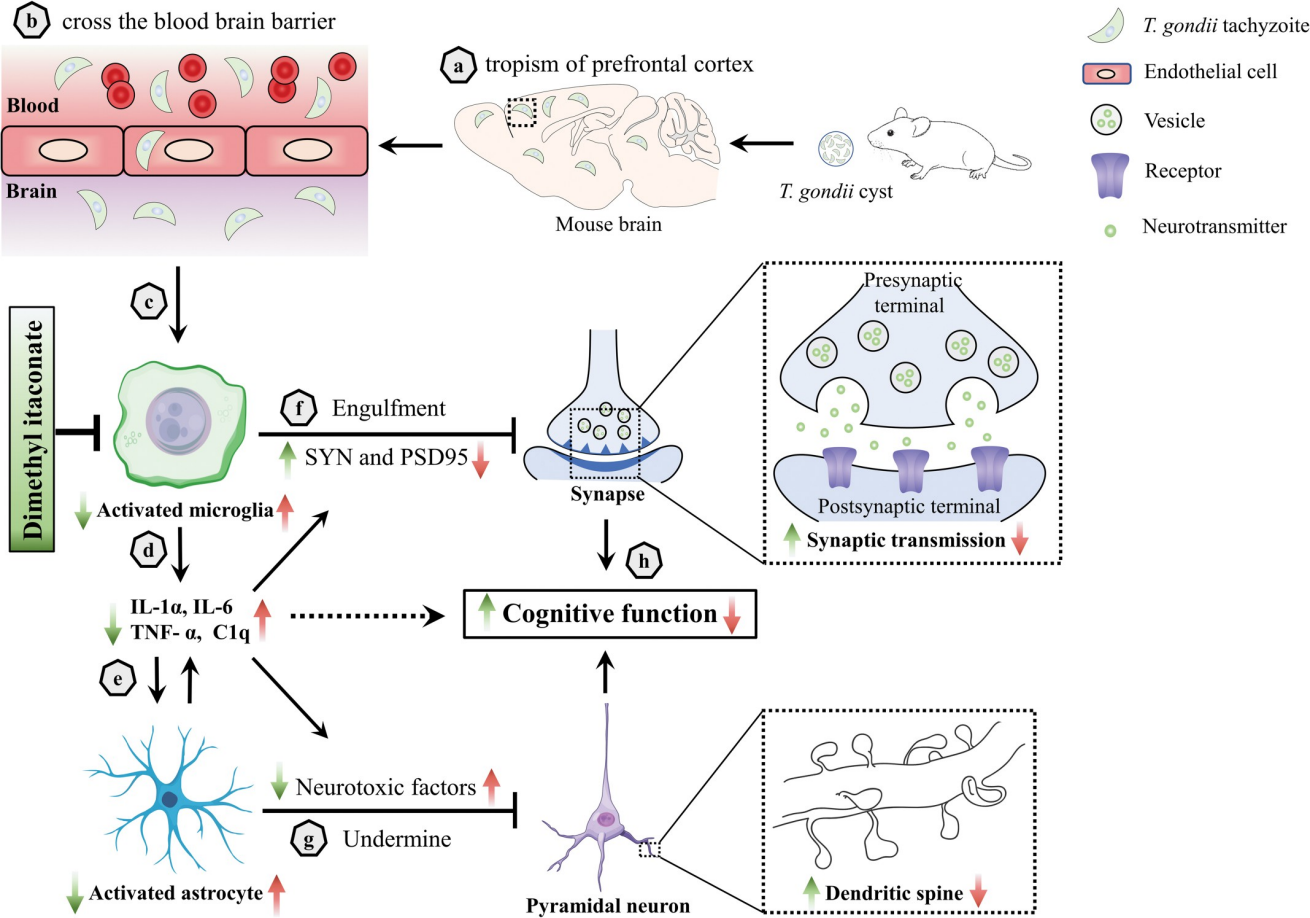
The schematic strategy of the prefrontal cortex-dependent cognitive deficits induced by *T*. *gondii* infection and the pro-cognitive effects of dimethyl itaconate. **a, b** *T*. *gondii* tends to the prefrontal cortex and enters the brain crossing the blood-brain barrier. **c, d** The invasion of *T*. *gondii* triggers the activation of microglia and the release of pro-inflammatory cytokines such as interleukin-1α (IL-1α), interleukin-6 (IL-6), tumor necrosis factor-α (TNF-α) and complement 1q (C1q). **e** Pro-inflammatory cytokines cause the activation of neurotoxic astrocytes, thereby amplifying neuroinflammation [24]. **f-h** Activated microglia engulf the plasticity-related protein like synaptophysin (SYN) and postsynaptic density 95 (PSD95), thereby impairing synaptic ultrastructure; neurotoxic astrocytes can undermine neuronal integrity. They together induce cognitive impairment. Red arrows represent the consequent events following *T*. *gondii* infection; Green arrows represent the pro-cognitive effect of dimethyl itaconate. Certain images in the figure were obtained from Scidraw (https://www.scidraw.io).

To the best of our knowledge, effective therapy against the cognitive deficits associated with *T*. *gondii* infection is not available, although the parasitic infection is well recognized to link with cognitive problems in animals and humans [8, 10, 13, 39]. Itaconate, a metabolite synthesized by the enzyme ACOD1, has recently emerged as a key regulator in macrophage function [28, 74]. In the present study, we showed that DI can significantly downregulate the expression of pro-inflammatory cytokines IL-6, IL-1β, TNF-α and CD86 (pro-inflammatory surface markers) in the prefrontal cortex of the infected mice, which indicates that DI can suppress the neuroinflammation induced by the infection. In line with this result, we observed an improved neuronal pathology in the DI-treated mice. A recent study uncovered that 4-octyl itaconate, a derivative of itaconate, can activate Nrf2 in microglia to protect against spinal cord injury in mice [75]. These results indicate that itaconate and its derivatives can exert a neuroprotective role via modulating neuroinflammation. In the last decades, it is increasingly recognized that neuroinflammation plays a crucial role in neurodegenerative diseases [17, 18, 25]. Our *in vivo* results confirmed that DI, a derivative of itaconate, suppresses the microglia-mediated neuroinflammation, thereby alleviating synaptic ultrastructure impairment and ameliorating cognitive deficits induced by chronic *T*. *gondii* infection (Fig 11). Recent studies uncovered that itaconate derivatives can ameliorate the cognitive impairment in the mouse model of AD [76] and alleviate β2-microglobulin-induced cognitive dysfunction [77]. Our previous study also showed that the derivative of itaconate could ameliorate the hippocampus-associated cognitive deficits in *T*. *gondii* infected mice [41]. Overall, these results suggest that itaconate may be a promising therapeutic molecular to treat neurodegenerative diseases including *T*. *gondii-*related cognitive deficits.

## Conclusion

The present study demonstrates that *T*. *gondii* infection induces the deficits of goal-directed cognitive behavior, accompanied by neuroinflammation, impaired neuronal integrity and synaptic ultrastructure in the prefrontal cortex of mice. Moreover, *T*. *gondii* infection significantly inhibited the expression of key genes associated with synapse plasticity, transmission, and behavior while upregulating the proinflammatory profiles. Interestingly, blockage of the Krebs cycle and disorder of the ACOD1-itaconate axis were identified to be the metabolic phenotypes in the prefrontal cortex post infection. Importantly, we provide evidence that DI can prevent and treat the deficits of the cognitive function induced by *T*. *gondii* infection. Overall, these findings lay a foundation for designing a novel therapy against *T*. *gondii*-related neurodegenerative diseases.

## Supporting information

S1 Table The qRT-PCR primer sequences used in this study. (TIF)

S2 Table GO analysis of prefrontal cortex from *T*. *gondii*-infected mice. Compilation of 23 significantly downregulated biological processes (n = 3).
(XLSX)

S3 Table GO analysis of prefrontal cortex from *T*. *gondii*-infected mice. Compilation of 672 significantly upregulated biological processes (n = 3).
(XLSX)

S4 Table KEGG pathway analysis of prefrontal cortex from *T*. *gondii*-infected mice. Compilation of 76 significantly enriched pathways (n = 3).
(XLSX)

S1 Fig GSEA analysis reveals the dysregulated synaptic transmission in the prefrontal cortex of *T*. *gondii*-infected mice. Enrichment plot of (**A**) GABA receptor activity, (**B**) Glutamatergic synapse, and (**C**) Dopaminergic synapse.
(TIF)

S2 Fig Dimethyl itaconate suppressed the *T*. *gondii*-induced activation of astrocytes in the prefrontal cortex of mice. **A** Representative immunofluorescent staining of the GFAP^+^ cells of astrocytes in the PFC (scale bar: 50 μm). The enlarged image captured from the box was marked with a solid line (scale bar: 10 μm). **B** Double immunofluorescence staining for GFAP (red) and IL-6 (green) in PFC of mice in Con+Veh, Con+DI, Tg+Veh, and Tg+DI group, white arrows represent Iba1^+^IL-6^+^ cells. Scale bar: 50 μm or 10 μm. **C** Quantification of GFAP^+^ astrocyte in the PFC (n = 3, 5 images per mouse). Con+Veh: control mice with Vehicle control treatment; Con+DI: control mice with DI treatment; Tg+Veh: *T*. *gondii* infected mice with Vehicle control treatment; Tg+DI: *T*. *gondii* infected mice with DI treatment. GFAP: glial fibrillary acidic protein; DAPI: nuclear staining; IL-6: interleukin-6. Values are presented as mean ± SEM. ***P <0.001.
(TIF)

S3 Fig Dimethyl itaconate upregulated the expression of synapse-related genes and decreased the cyst number in the brain of *T*. *gondii* infected mice. **A, B** The mRNA expression of SYN and PSD95 in the prefrontal cortex (n = 4–6). **C** The Cyst enumeration in the brain from treated and untreated group mice (n = 4).
(TIF)
