# Supplementary figures and images for: Dimethyl itaconate ameliorates the deficits of goal-directed behavior in Toxoplasma gondii infected mice
Source: PLoS Negl Trop Dis. 2023 May 31;17(5):e0011350. doi: 10.1371/journal.pntd.0011350 (PMC10231842; doi:10.1371/journal.pntd.0011350)

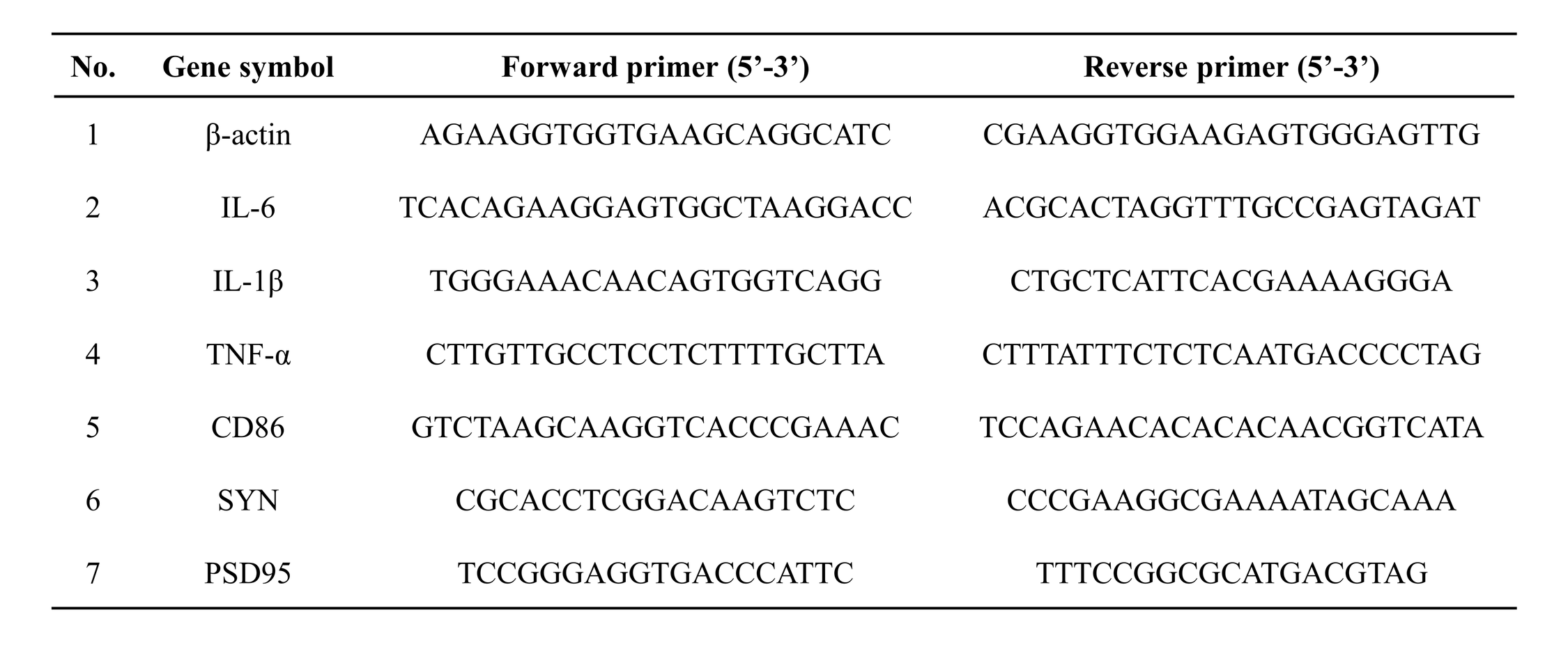

Supplement: S1 Table — (TIF) [file pntd.0011350.s001.tif]

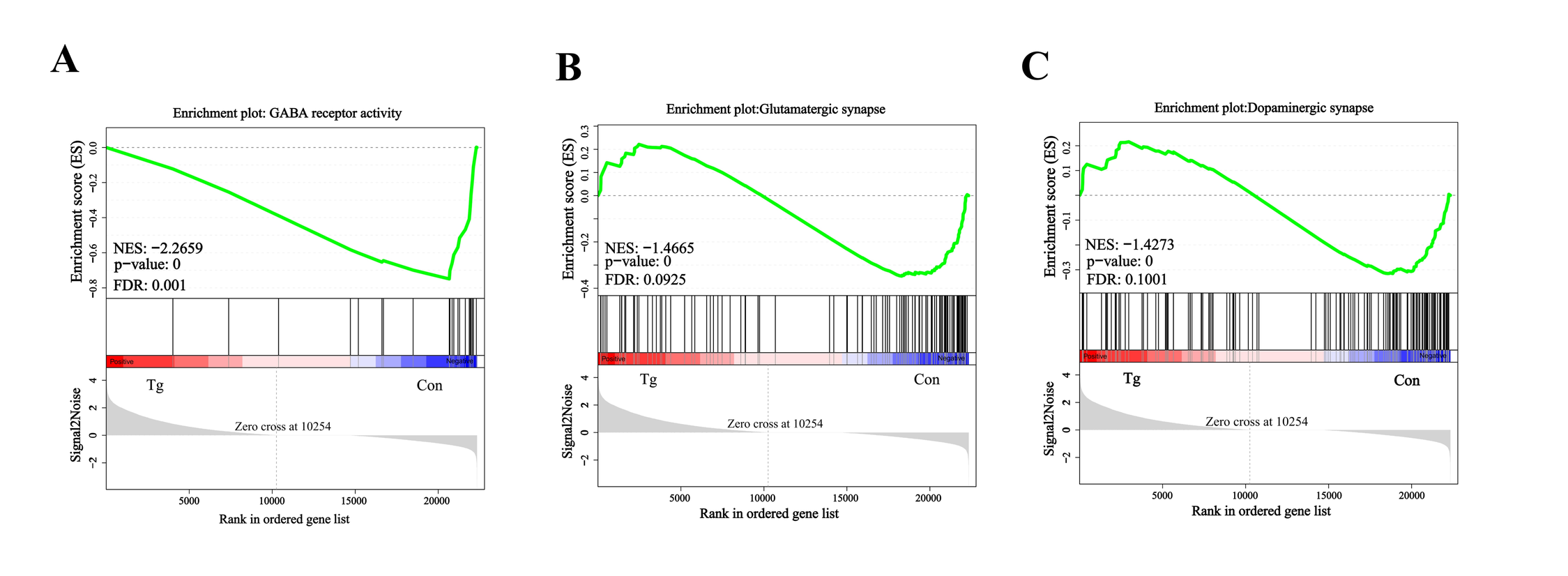

Supplement: S1 Fig — Enrichment plot of (A) GABA receptor activity, (B) Glutamatergic synapse, and (C) Dopaminergic synapse. (TIF) [file pntd.0011350.s005.tif]

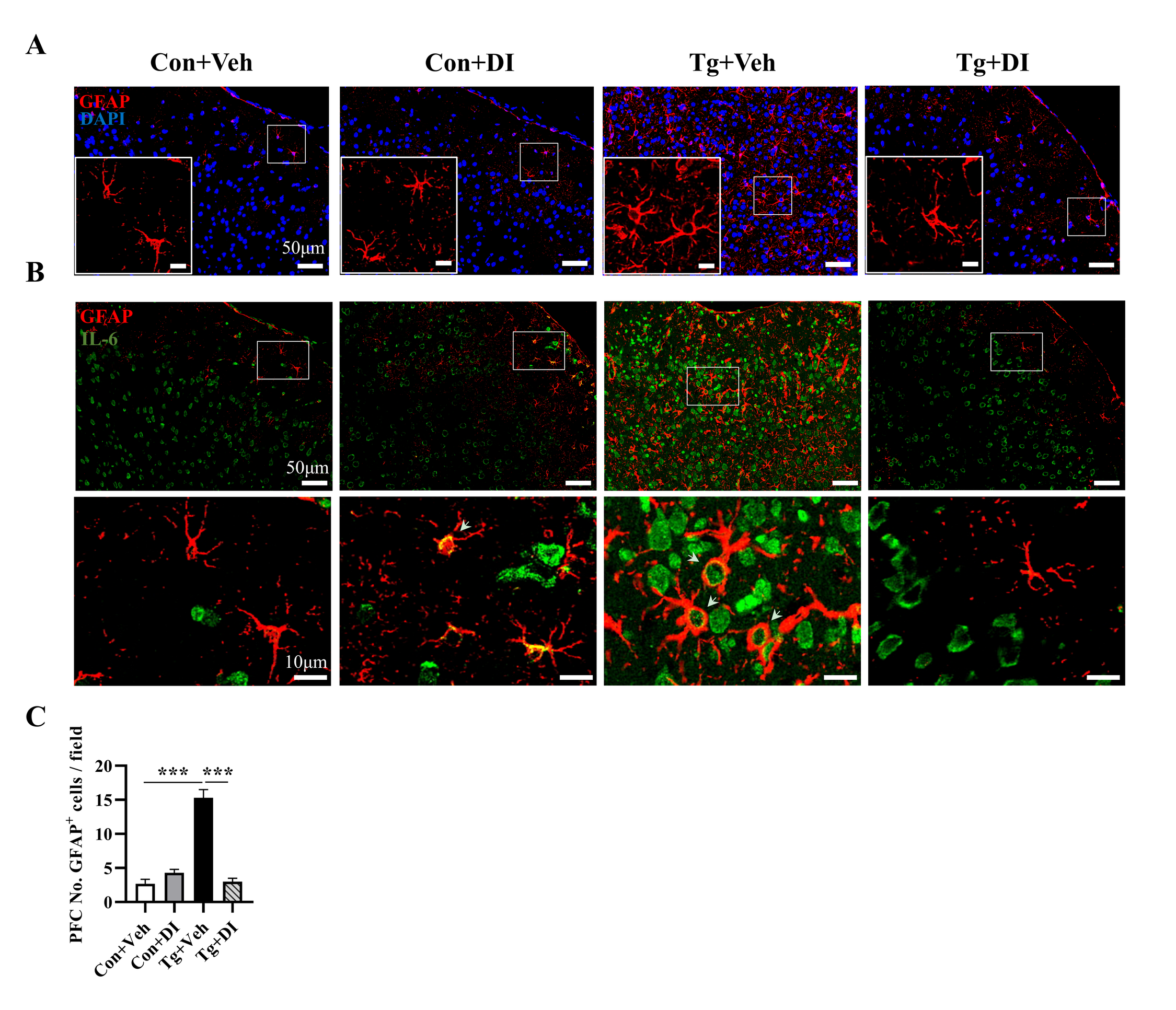

Supplement: S2 Fig — A Representative immunofluorescent staining of the GFAP+ cells of astrocytes in the PFC (scale bar: 50 μm). The enlarged image captured from the box was marked with a solid line (scale bar: 10 μm). B Double immunofluorescence staining for GFAP (red) and IL-6 (green) in PFC of mice in Con+Veh, Con+DI, Tg+Veh, and Tg+DI group, white arrows represent Iba1+IL-6+ cells. Scale bar: 50 μm or 10 μm. C Quantification of GFAP+ astrocyte in the PFC (n = 3, 5 images per mouse). Con+Veh: control mice with Vehicle control treatment; Con+DI: control mice with DI treatment; Tg+Veh: T. gondii infected mice with Vehicle control treatment; Tg+DI: T. gondii infected mice with DI treatment. GFAP: glial fibrillary acidic protein; DAPI: nuclear staining; IL-6: interleukin-6. Values are presented as mean ± SEM. ***P <0.001. (TIF) [file pntd.0011350.s006.tif]

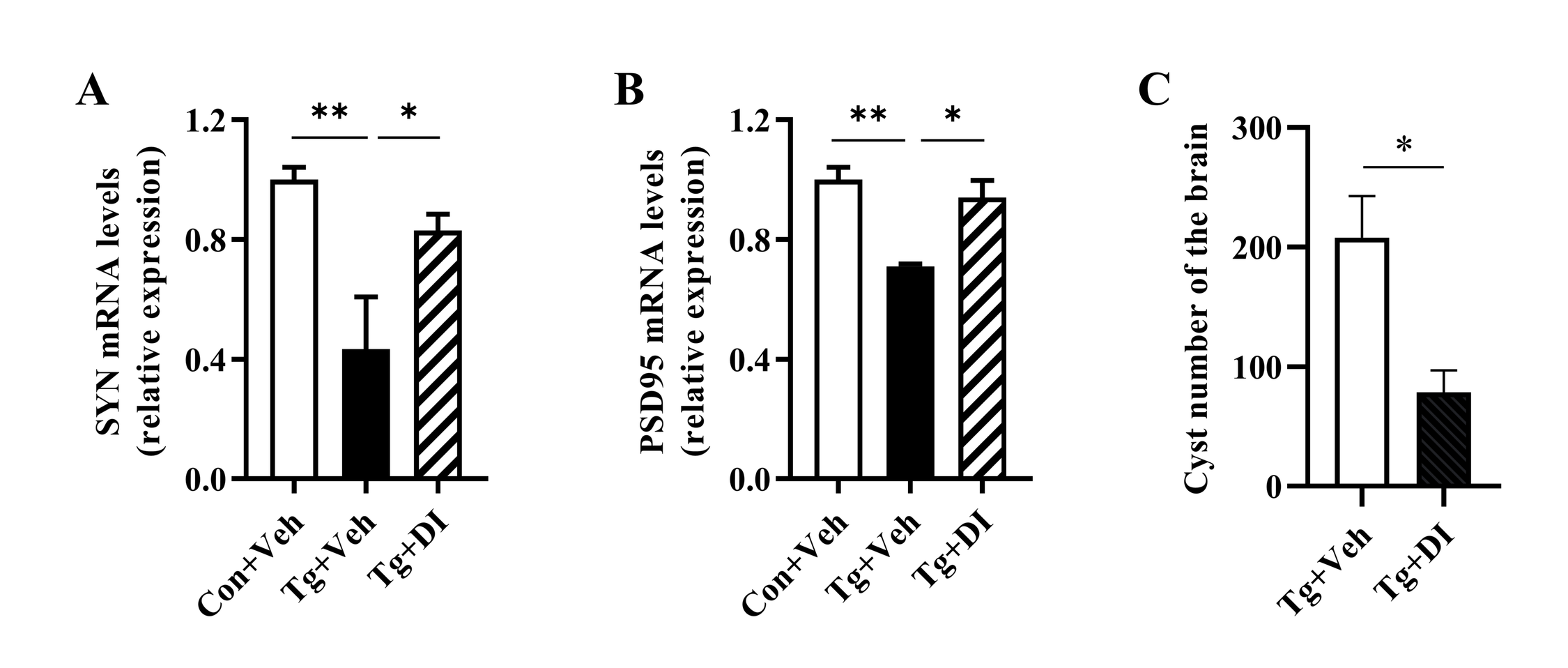

Supplement: S3 Fig — A, B The mRNA expression of SYN and PSD95 in the prefrontal cortex (n = 4–6). C The Cyst enumeration in the brain from treated and untreated group mice (n = 4). (TIF) [file pntd.0011350.s007.tif]
